# Interface Construction via Scavenging Oxygen Species Enables High‐Performance Li‐Rich Layered Oxide Cathode

**DOI:** 10.1002/advs.202524384

**Published:** 2026-01-21

**Authors:** Jiahe Chen, Haoran Ma, Jiajia Huang, Hongbo Wu, Zhen Yang, Chenchen Li, Zhijun Wu, Jingwei Zhao, Min Zhu, Jun Liu

**Affiliations:** ^1^ Guangdong Provincial Key Laboratory of Advanced Energy Storage Materials School of Materials Science and Engineering South China University of Technology Guangzhou China; ^2^ Institute of Science and Technology for New Energy Xi'an Technological University Xi'an China; ^3^ Guangzhou Tinci Materials Technology Co. Ltd. Guangzhou China

**Keywords:** a robust CEI film, high‐energy‐density, lithium‐rich layered oxide, nucleophilic attack, reactive oxygen species

## Abstract

Lithium‐rich layered oxide (LRLO) is a promising material for high‐energy‐density lithium‐ion batteries (LIBs), yet its practical application is hindered by the nucleophilic attack of reactive oxygen species (ROS) on carbonate electrolytes, inducing severe interfacial incompatibility. Herein, a LiPF_6_‐based carbonate electrolyte modified by dual B/P‐containing additives with oxygen scavenging capability is developed to mitigate these issues. The designed electrolyte enables efficient ROS scavenging and in situ formation of a robust cathode/electrolyte interface (CEI) on the LRLO surface. The designed electrolyte not only enhances the reversibility of anionic redox reactions (ARRs) but also suppresses electrolyte decomposition and transition metal dissolution. Electrochemical tests demonstrate that the Li//LRLO cell with a high mass loading of 15 mg cm^−2^ retains 94.4% of its initial capacity after 200 cycles, while the 4.5 Ah graphite//LRLO pouch cell exhibits a higher energy density of 282.5 Wh kg^−1^ with stable cycling over 50 cycles. This work offers a viable strategy for interface engineering of LRLO‐based cathodes, paving the way for the development of high‐performance LIBs with long‐term cyclic stability.

## Graphical abstract

To inhibit electrolyte decomposition by nucleophilic attack in carbonate electrolyte, LiBOB and LiDFOP with strong oxygen scavenging ability are selected to quench reactive oxygen species and following construct a robust CEI film on the LRLO cathode. The electrochemical performance of the LRLO cathode is largely improved, attributed to decomposition suppression and structural stability, when cycled in C‐BP electrolyte.

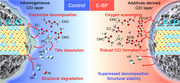

## 1 Introduction

Rechargeable lithium‐ion batteries have become the dominant power source for portable electronics and are rapidly penetrating electric‐vehicle and grid‐storage markets owing to their long‐term cycling stability and low self‐discharge [1, 2, 3]. With the rapid development of energy storage systems, the existing LIBs are increasingly unable to meet the urgent demand for higher energy density [4, 5, 6]. Although researchers have made significant progress in optimizing electrode structures and electrolyte systems, LIBs still face key challenges in pursuing high energy density. The actual capacity of traditional cathode materials (LiCoO_2_, LiNi_0.8_Co_0.1_Mn_0.1_O_2_, and LiFePO_4_) approaches the theoretical limit, restricting the overall energy density improvement [7, 8, 9]. Additionally, the matching of high‐voltage cathodes with electrolytes causes interface instability, which poses potential safety hazards [10, 11].

To satisfy the ever‐increasing energy demand of next‐generation devices, cathode materials that can deliver high specific capacities over 250 mAh g^−1^ are urgently required [12, 13]. Li‐rich layered oxide materials (xLi_2_MnO_3_·(1‐x)LiTMO_2_, TM = Ni, Co, Mn) have emerged as a highly promising candidate for next‐generation and high‐energy‐density LIB cathodes [14, 15, 16]. Compared to conventional cathode materials, LRLO materials exhibit a distinct advantage in terms of high specific capacity. The exceptional capacity arises from the unique dual redox mechanism involving both cationic (such as Ni^2+^/Ni^3+^/Ni^4+^, Co^3+^/Co^4+^) and anionic redox reactions (ARRs, O^2−^/O^n−^, n<2) during the charge/discharge process, a feature that distinguishes them from traditional cathodes relying solely on cationic redox [17, 18, 19]. Nevertheless, LRLO cathodes face several critical technical challenges that have impeded their practical application in commercial LIBs [20]. During repeated charge/discharge cycles, LRLO cathodes undergo a series of intricate structural and chemical transformations that collectively lead to capacity decay [13, 21, 22]. During the ARRs, reactive oxygen species, including singlet oxygen (^1^O_2_) and peroxo‐like species (O^n−^, n<2), are released from the LRLO lattice, which are prone to reacting with the components of the conventional electrolyte [23, 24, 25]. This reaction triggers continuous electrolyte decomposition, producing gaseous products (such as O_2_, CO_2_, CO) and forming an unstable interface [26, 27]. The unstable interface lacks long‐term structural integrity and tends to crack and reform during cycling, failing to effectively isolate the cathode from the electrolyte. This leads to persistent consumption of active lithium ions, gradual loss of active cathode materials, and a significant increase in interfacial impedance. Additionally, during cycling, LRLO cathodes undergo a gradual structural change from an original layered structure to a spinel or rock‐salt structure [28, 29]. This transformation further impairs their electrochemical performance and rate capability, leading to an accelerated decline in cycle life.

To mitigate the cycle instability issues of LRLO cathodes, researchers have dedicated extensive efforts to developing various modification strategies in electrolyte engineering [30, 31, 32]. Among these approaches, additive modification in the electrolyte system has gained considerable interest due to its simplicity, cost‐effectiveness, and its ability to simultaneously tackle interfacial issues across the entire cathode surface [33, 34]. To deal with the nucleophilic attack from ROS, the effective and direct strategy is to introduce an additive with strong oxygen scavenging capability. Kang et al. introduced dimethyl sulfide (DMS) as an additive in carbonate electrolyte to enhance the interfacial stability of high‐voltage LiNi_0.8_Co_0.1_Mn_0.1_O_2_ cathodes [35]. The highest occupancy molecular orbital (HOMO) of the DMS additive is positioned at a higher energy level than solvents, indicating that it is more likely to undergo oxidation to participate in CEI formation. Additionally, DMS can effectively neutralize superoxide radical from LiNi_0.8_Co_0.1_Mn_0.1_O_2_, thereby suppressing further side reactions. Another effective additive with a B atom has been demonstrated that this kind of additive can bond with ROS by forming B‐O bonds, effectively anchoring transition metal ions (TMs) and forming a stable CEI film [36, 37, 38]. P‐containing additives with a low‐electron‐density center, especially phosphite ester with P(III), potentially accept ROS with nucleophilic characteristics to form P ═ O bonds [39, 40]. Wei et al. developed a carbonate electrolyte by incorporating dual lithium difluoro(oxalato)borate (LiDFOB) and tri(trimethylsilyl) phosphite (TMSPi) additive (EEDB‐TMSPi) [41]. The addition of dual additives tailored the solvation structure with enhanced desolvation property and high oxidation‐resistance. The EEDB‐TMSPi enables an excellent operation of Li//LRLO in a wide temperature from −20 to 55°C.

In this work, we incorporated lithium bis(oxalate)borate (LiBOB) and lithium difluorobis(oxalato) phosphate (LiDFOP) additives into a lithium hexafluorophosphate (LiPF_6_)‐based carbonate electrolyte (C‐BP) to scavenge oxygen species and convert a robust CEI film on the LRLO surface. The electrolyte with LiBOB and LiDFOP exhibits electrochemical oxidation stability in applied voltage and enables the reversible ARRs through oxygen scavenging and robust CEI formation. Electrolyte decomposition is mitigated by protecting solvent molecules from ROS and reducing the hydrolysis of LiPF_6_. As a result, the Li//LRLO cell with a high mass loading of 15 mg cm^−2^ demonstrates excellent capacity retention of 94.4% after 200 cycles. Additionally, the 4.5 Ah‐class graphite//LRLO pouch cell achieves an energy density of 282.5 Wh kg^−1^ with sustained cycling performance over 50 cycles.

## 2 Results and Discussion

### 2.1 Physicochemical Properties of the Designed Electrolyte

**FIGURE 1:**
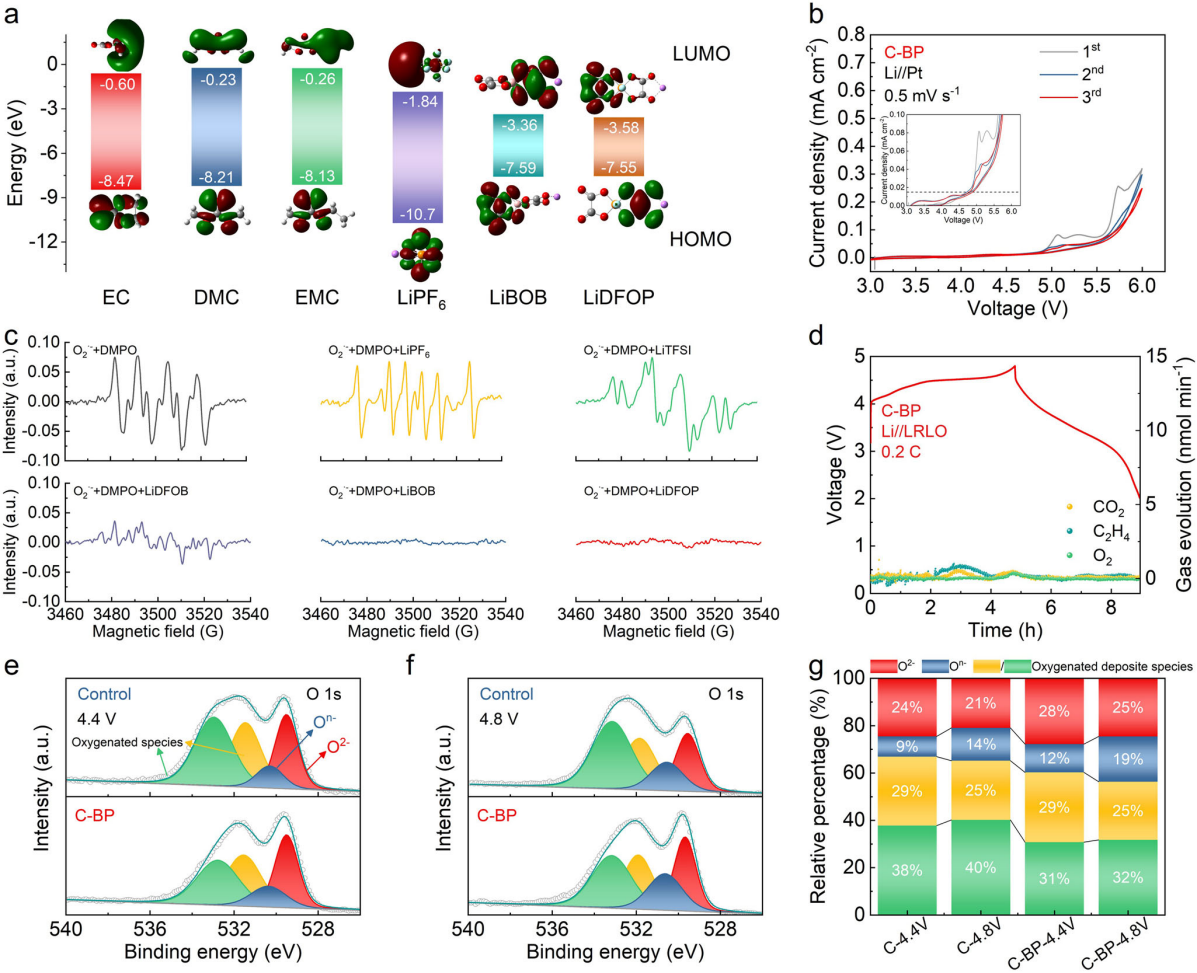
(a) HOMO and LUMO energy levels of various Li salts and solvents. (b) Cyclic voltammetry profiles of Li//Pt cells with C‐BP electrolytes at the scanning rate of 0.5 mV s^−1^. (c) EPR results of the mixed solution (KO_2_+DMPO+AN) with LiPF_6_, LiTFSI, LiDFOB, LiBOB, and LiDFOP. (d) Gas analysis for Li//LRLO cell in C‐BP electrolyte during first charge and discharge at 0.2 C. The evolution of O 1s XPS spectra for the LRLO cathode in the control and C‐BP electrolytes during the first cycle at (e) 4.4 V and (f) 4.8 V. (g) The corresponding relative proportion statistics of different species obtained from the O 1s XPS spectra.

To investigate the effects of LiBOB and LiDFOP additives on the interfacial formation and irreversible oxygen inhibition, 0.1 M concentrations of LiBOB and LiDFOP additives were added in a carbonate electrolyte (1 M LiPF_6_ in EC:DMC:EMC with a volume ratio of 1:1:1), named C‐BP electrolyte. For comparison, the control electrolyte was prepared by dissolving 1.2 M LiPF_6_ in EC:DMC:EMC with a volume ratio of 1:1:1. In Figure 1a, the energy levels of the HOMO and the lowest unoccupied molecular orbital (LUMO) of lithium salts and solvents were determined by density functional theory (DFT). The lithium salts (LiPF_6_, LiBOB, and LiDFOP) exhibit relatively lower LUMO values, reflecting their ability to prompt the formation of a solid electrolyte interface (SEI) on the anodes. While the LiBOB and LiDFOP additives show higher HOMO values compared to other components in the electrolyte, suggesting that they prior to undergo oxidation decomposition during the charge process. The linear sweep voltammetry (LSV) profiles show that the oxidation of LiBOB and LiDFOP occurs at the voltages of around 4.4 and 4.6 V vs. Li/Li^+^, respectively (Figure S1). The peaks at around 4.5 V vs. Li/Li^+^ in the LSV curve of C‐BP electrolyte indicate the electrochemical decomposition of both additives from electron attack. Therefore, both additives potentially participate in the ARRs at the 4.5 V plateau of LRLO cathodes to scavenge ROSs. To determine the oxidation stability of the as‐prepared electrolytes, cyclic voltammetry (CV) tests of Li//Pt with different electrolytes were performed at the scanning rate of 0.5 mV s^−1^. The control electrolyte demonstrates strong oxidation resistance, showing an oxidation window of 5.45 V during the initial scan from the open circuit voltage to 6 V vs. Li/Li^+^ (Figure S2). Nevertheless, the subsequent CV curves reveal a decline in oxidation resistance, with the oxidation window shrinking to 3.44 V. This indicates that some side products generated during the initial scan trigger severe oxidation decomposition of the electrolyte. In the C‐BP electrolyte, severe solvent decomposition can be effectively suppressed, with no apparent shrinking of the CV curves. Additionally, the oxidation voltage of the C‐BP electrolyte stabilizes at 4.89 V (Figure 1b).

Specially, anionic redox reaction in the LRLO cathodes contributes to a high capacity, which requires increasing the cut‐off voltage up to 4.6 V [20]. During the initial activation of the Li_2_MnO_3_ in the LRLO cathodes, some components in the electrolyte will undergo nucleophilic attack by reactive oxygen species [42]. Therefore, the electrolyte suffers from two oxidation processes triggered by either the applied potential or reactive oxygen species during the cycling of the LRLO cathodes. For illuminating the oxygen scavenging ability of different Li salts, the radical trapping experiment was further performed to verify the strong scavenging effect of LiBOB and LiDFOP toward O_2_·^−^ radical. As illustrated in Figure 1c, the active signals in electron paramagnetic resonance (EPR) are attributed to O_2_·^−^ radical trapped by 5‐5‐dimethyl‐1‐1pyrroline N‐oxide (DMPO). The introductions of inorganic salt LiPF_6_ and organic salt lithium bis(trifluoromethanesulphonyl)imide (LiTFSI) can not quench the O_2_·^−^ radical with the presenting active EPR signals. However, three kinds of oxalate salts (lithium difluoro(oxalato)borate (LiDFOB), LiBOB, and LiDFOP) exhibit excellent oxygen scavenging properties in the presence of KO_2_. In comparison to LiDFOB, both LiBOB and LiDFOP effectively neutralize all O_2_·^−^ radicals, resulting in the absence of any EPR signals. Therefore, LiBOB and LiDFOP salts are selected to effectively scavenge oxygen species and improve the electrochemical performance of LRLO materials. Furthermore, in situ differential electrochemical mass spectrometry (DEMS) was conducted to detect gas evolution during the electrochemical cycle. The increasing signals of the products (e.g., CO_2_ and C_2_H_4_) derived from electrolyte decomposition can be classified into three distinct regions: (i) catalytic decomposition occurring on the transition metal surface, (ii) ARRs, and (iii) high‐voltage decomposition (Figure S3). Notably, numerous CO_2_ and C_2_H_4_ gases are released in the ARRs, indicating the control electrolyte suffers from severe nucleophilic attack from active oxygen species. In comparison, slight electrolyte decomposition and reduced O_2_ release were detected in the C‐BP electrolyte, which inhibits the oxygen loss and boosts the reversibility of the ARRs (Figure 1d).

Ex situ X‐ray photoelectron spectroscopy (XPS) measurements were performed to investigate the evolution of the electronic structure of oxygen and CEI film formation during the initial electrochemical cycles. The pristine LRLO cathode exhibits a prominent lattice oxygen (O^2−^) peak at 529.6 eV in O 1s XPS spetra, while two additional peaks are assigned to impurity on the LRLO cathodes (Figure S4). The control electrolyte shows high oxidation resistance from applied potential at the initial CV scans, while the carbonate component in the control electrolyte suffers from nucleophilic attack from oxygen species of LRLO cathodes. When charged to 4.4 V, a new peak corresponding to peroxo‐like species (O^n−^, n<2) appears at 530.3 eV in the O 1s spectra of the LRLO cathode in both the control and C‐BP electrolyte (Figure 1e). This suggests that lattice oxygen is partially converting into highly reactive oxygen species. As the voltage is increased to 4.8 V, more lattice O^2−^ is oxidized to O^n−^ for charge compensation in LRLO cathodes (Figure 1f,g). In the control electrolyte, the proportion of O^n−^ component rises from 9% at 4.4 V to 14% at 4.8 V. Nevertheless, the proportion of O^n−^ component remains higher value in the C‐BP electrolyte both at 4.4 and 4.8 V. These results suggest that the irreversible oxygen loss, including O_2_ emission and nucleophilic electrolyte decomposition, occurs in the control electrolyte when used in LRLO cathodes. The additives in C‐BP electrolyte improve the reversibility of the anionic oxygen reaction and prevent oxygen loss. The mechanism of electrolyte decomposition can be deduced by the analysis of side products on the LRLO cathodes. The P‐F and LiP_x_O_y_F_z_ species appeared in the P 2p XPS spectra of the control electrolyte are ascribed to the decomposition of LiPF_6_ (Figure S5a). And previous research has demonstrated that the oxidation decomposition of carbonate solvents promotes LiPF_6_ hydrolysis under high voltage conditions or nucleophilic attack. Thereby, the progressive LiPF_6_ hydrolysis renders to produce more P‐F species when the voltage is increased to 4.8 V (Figure S5b). In contrast, the LiDFOP additive, with its higher HOMO energy level, can be preferentially oxidized at the high‐voltage cathodes. The P‐containing species observed in the XPS spectra should be attributed to the decomposition of LiDFOP. The content of LiP_x_O_y_F_z_ shows an increasing trend when the voltage increases from 4.4 to 4.8 V (Figure S5a,b). Additionally, a greater amount of LiF component is generated on the LRLO surface at 4.8 V (Figure S5c,d). These findings suggest that the LiDFOP additive is able to scavenge oxygen species to form a robust CEI film enriched with LiP_x_O_y_F_z_ and LiF. Similarly, the LiBOB additive, with an electron‐deficient B center, exhibits oxygen scavenging capability and contributes to the CEI formation, leading to an increase in Li_x_B_y_O_z_ content at 4.8 V (Figure S5e).

In order to reveal the boosting oxygen reversibility in the LRLO cathodes with C‐BP electrolyte, in situ electrochemical impedance spectra (EIS) were tested. The converted distribution of relaxation time (DRT) results can distinguish the impedance contribution in an electrochemical system [43, 44]. Three main kinetic processes can be identified as the impedance of CEI (R_CEI_, τ<10^−2^), charge transfer (R_ct_, 10^−2^<τ<10), and diffusion (R_diffusion_, τ>10) (Figure S6). In the control electrolyte, a significant increase in the R_ct_ contribution is observed in ARRs above 4.5 V during the initial activation, which is attributed to continuous oxygen loss (Figure S6a). The side products derived from the nucleophilic attack result in an increase in the impedance of both R_CEI_ and R_ct_ during the subsequent charge/discharge process. In contrast, the C‐BP electrolyte offers advantages by decreasing the impedance of CEI and charge transport during activation, which signifies an improvement in the kinetics of ARRs. The small R_CEI_ and R_ct_ observed in the subsequent cycle contribute to the preservation of oxygen reversibility in LRLO cathodes. To further determine the parasitic reactions between electrolytes and LRLO cathodes, the leakage current was recorded using Li//LRLO half cells after the initial activation process (Figure S7). By setting fixed floating charge voltages at 4.8, 4.9, and 5.0 V for 24 h, the leakage currents of the cell in C‐BP electrolyte were measured to be 2.8, 3.4, and 6.2 µA, respectively, smaller than that in the control electrolyte (9.3, 11.5, and 13.0 µA). The results demonstrate the remarkable electrochemical stability of the C‐BP electrolyte at high voltages, attributed to the efficient CEI film formed on the LRLO cathodes.

To determine the activation energies (*E_a_*) of Li^+^ desolvation, EIS data of Li//Li symmetric cells with different electrolytes were recorded at different temperatures. As shown in Figure S8, the *E_a_* of the C‐BP electrolyte (64.5 kJ mol^−1^) is lower than that of the control electrolyte (73.8 kJ mol^−1^), indicating that the addition of additives lowers the desolvation barrier for Li^+^ and facilitates the desolvation process of Li^+^. In Figures S9 and S10, the involvement of the additives results in a slight decrease in the ion conductivity and a slight increase in the density of the C‐BP electrolyte (11.6 mS cm^−1^, 1.268 g mL^−1^), compared to the control electrolyte (12.7 mS cm^−1^, 1.250 g mL^−1^). However, the C‐BP electrolyte exhibits a higher Li^+^ transfer number (0.63) than the control electrolyte (Figure S11). The wettability of the C‐BP exhibits a smaller contact angle with the separator than that of the control electrolyte (Figure S12).

### 2.2 Electrochemical Performance of LRLO Cathode

**FIGURE 2:**
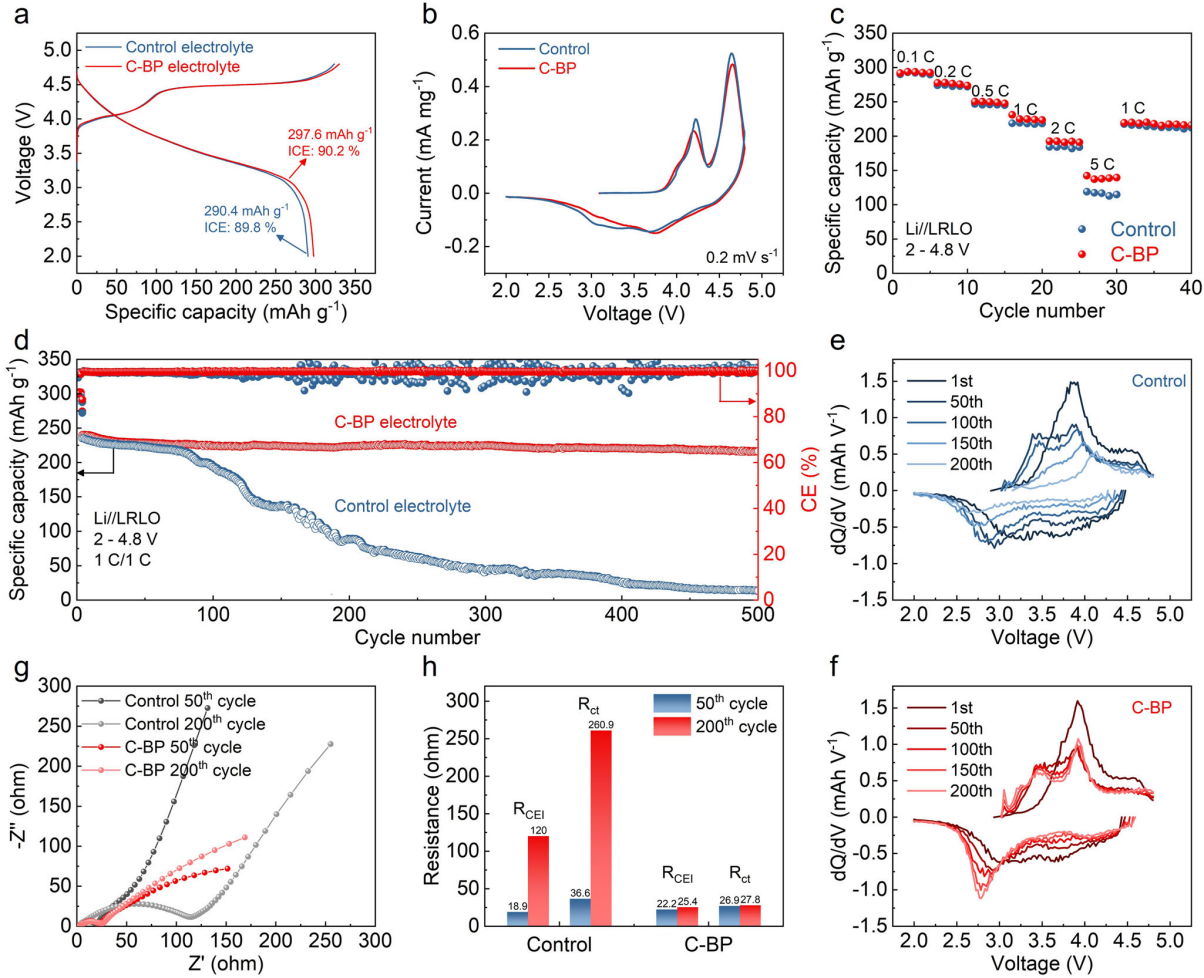
(a) Initial charge/discharge curves of Li//LRLO cells in the control and C‐BP electrolytes obtained at 0.1C in the voltage window of 2–4.8 V. (b) Cyclic voltammetry curves of Li//LRLO cells with the control and C‐BP electrolytes at the scanning rate of 0.2 mV s^−1^. (c) Rate capability of Li//LRLO cells in the control and C‐BP electrolytes. (d) Cycling performance of Li//LRLO cells cycled in the control and C‐BP electrolytes at 1C within the electrochemical window of 2–4.8 V. The corresponding dQ/dV profiles of selected cycles for the LRLO half cells in the (e) control and (f) C‐BP electrolytes. (g) EIS spectra and (h) the impedance comparison of the R_CEI_ and R_ct_ of LRLO cells cycled in the control and C‐BP electrolytes at 50 and 200 cycles.

Li//LRLO half cells were investigated to evaluate the effectiveness of LiBOB and LiDFOP additives on enhancing the electrochemical performance of LRLO cathodes. The typical charge/discharge curves of the half cells in different electrolytes at 0.1C (1C = 250 mAh g^−1^) are illustrated in Figure 2a. The charge curves can be divided into three distinct sections: (i) the cationic redox reaction of Ni and Co occurring below 4.5 V; (ii) the extended charging plateau above 4.5 V related to the Li_2_MnO_3_ activation; and (iii) electrochemical decomposition of electrolyte between 4.6 and 4.8 V. The Li//LRLO half cell with the C‐BP electrolyte delivers a high discharge specific capacity of 297.6 mAh g^−1^ with a high initial coulombic efficiency (ICE) of 90.2 %. In contrast, the control electrolyte with poor oxidation stability triggers severe electrolyte decomposition in the initial activation process of the LRLO cathode with a low discharge capacity (290.4 mAh g^−1^) and ICE (89.8 %). CV tests were performed to further investigate the impact of additives on the redox behavior of the LRLO cathodes in different electrolytes. As illustrated in Figure 2b, the oxidation peaks observed around 4.20 V during the first cycle are attributed to the cationic redox reaction, involving Ni^2+^/Ni^4+^ and Co^3+^/Co^4+^. Additionally, as the scan progresses to higher voltage, the extraction of Li^+^ and the escape of O^2−^ from the Li_2_MnO_3_ phase lead to the formation of additional oxidation peaks at around 4.66 V. The slight upshift of the cationic‐redox‐related peak and the enhanced intensity of the anionic‐redox‐related peak illustrate a slow kinetics of Li^+^ extraction from layered LiTMO_2_ and severe electrolyte decomposition triggered by active oxygen species in the control electrolyte.

The rate performance is crucial for the practical use of LIBs, as the charging time and discharging capacity of LIBs depend on the rate at which Li^+^ ions penetrate the CEI film and Li^+^ ions are intercalated/deintercalated from the LRLO cathodes. The Li//LRLO cell in the C‐BP electrolytes exhibits better rate performances at different current densities over 1C under a 4.8 V cut‐off voltage (Figure 2c; Figure S13). The cell with the control electrolyte delivers only 116.1 mAh g^−1^ discharge capacity at 5C, while the discharge capacity of the cell with the C‐BP electrolyte remained at 139.1 mAh g^−1^. To analyze the electrochemical reaction kinetics of LRLO cathodes in C‐BP electrolyte, CV was conducted at various scan rates using Li//LRLO cells. The linear correlation observed between the sweep rate (v^1/2^) and peak current reflects the electrochemical kinetics in LRLO cathodes. Especially, the slope of this relationship is directly proportional to the diffusion coefficients. The slope of the oxidation and reduction peaks in the C‐BP electrolyte is larger than that in the control electrolyte, indicating the Li^+^ ions can diffuse more easily across the surface interphase of the LRLO cathode in C‐BP electrolyte (Figure S14). The effect of the C‐BP electrolyte on enhanced cyclic stability was determined by Li//LRLO cells with a voltage window of 2–4.8 V at 1C (Figure 2d). The charge/discharge curves of LRLO in the control and C‐BP electrolyte are illustrated in Figure S15. The cell with the control electrolyte exhibits poor stability during long‐term cyclic performance with a severe capacity decay after 100 cycles. Notably, the LRLO cathodes cycled in the C‐BP electrolyte can maintain a high capacity of 216.0 mAh g^−1^ after 500 cycles at 1C, with a highest capacity retention of 90.0%, while those for the cells with the control electrolyte are only 136.0 mAh g^−1^ and 57.9% of the control electrolyte after 150 cycles.

The dQ/dV profiles obtained from different charge/discharge curves reflect significant information on the redox contribution and structural evolution of the LRLO cathodes (Figure 2e,f). The typical peaks located at around 3.90 and 3.67 V correspond to the oxidation and reduction process of the Ni^2+^/Ni^4+^ and Co^3+^/Co^4+^ redox reaction, respectively. Additionally, the redox processes of the Mn^3+^/Mn^4+^ appear at 3.46 V in the charging process and at 2.98 V in the discharging process. As shown in Figure 2e, the LRLO cathode cycled in the control electrolyte exhibits significant decreases in peak intensity and peak shifts to high voltage for the oxidation processes of Ni and Co. Correspondingly, the peaks of the oxidation and reduction of Mn gradually disappear following the progressing cycles. These changes of redox peaks in the control electrolyte indicate the severe TMs dissolution and or the significant structural transformation in the LRLO cathodes. Conversely, the peaks of the oxidation and reduction of TMs ions can be well preserved in the LRLO cathodes cycled in the C‐BP electrolyte, illustrating the suppressed structural evolution enabled by the inhibition of electrolyte decomposition at high voltage (Figure 2f). Additionally, the interfacial resistance of LRLO cathodes in different electrolytes was evaluated using an electrochemical impedance spectrum (EIS) at different cycles. As depicted in Figure 2g,h, both the CEI resistance (R_CEI_) of the high‐frequency semicircle and the charge transfer resistance (R_ct_) in the middle‐frequency region experience a significant increase after 200 cycles for the LRLO cathode cycled in the control electrolyte. However, the R_CEI_ and R_ct_ values of the LRLO cathode in C‐BP electrolyte remain relatively stable at 25.4 and 27.8 Ω, respectively, even after 200 cycles. It indicates that the use of C‐BP electrolyte in the LRLO cathode effectively suppresses electrolyte decomposition and enhances structural stability. The stable interphase formed in the C‐BP electrolyte plays a key role in delivering the excellent cycling performance.

**FIGURE 3:**
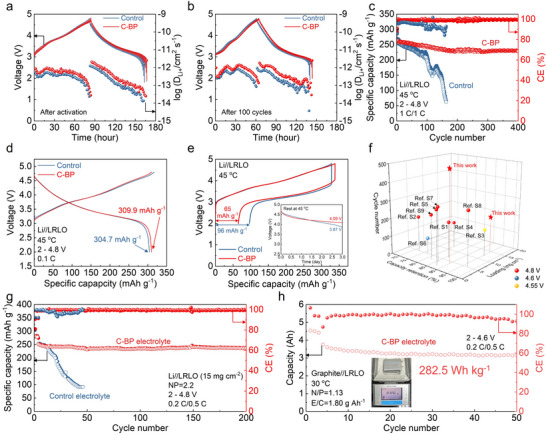
Charge/discharge voltage profiles of GITT measurements for the Li//LRLO cells with the control and C‐BP electrolytes after (a) activation and (b) 100 cycles. All cells were cycled at 0.1C for 10 min followed by 1 h relaxation. (c) High‐temperature cycling stability of Li//LRLO cells in the control and C‐BP electrolytes at 1C and (d) corresponding charge/discharge curves of Li//LRLO at 0.1C under 45 °C. (e) Charge/discharge curves of Li//LRLO cells after resting at 45 °C for 72 h (inset: the corresponding open‐circuit voltage behavior). (f) Comparisons of the electrochemical performance of LRLO cathodes in different high‐voltage electrolytes. (g) Electrochemical performance of high mass loading LRLO (15 mg cm^−2^) with a limited lithium anode (N/P ratio = 2.2) in the control and C‐BP electrolytes at 0.2C charge/0.5C discharge. (h) Cycling stability of pouch‐type graphite//LRLO full cell in the C‐BP electrolyte with N/P ratio of 1.13 and E/C ratio of 1.8 g Ah^−1^ at 0.2C charge/0.5C discharge.

To elucidate the protective effect of additive‐derived CEI on maintaining the structural integrity of LRLO cathodes, galvanostatic intermittent titration technique tests (GITT) were applied to explore kinetic behaviors of the LRLO cathodes at different cycles. As illustrated in Figure 3a, the LRLO cathodes cycled in both control and C‐BP electrolytes exhibit similar charge/discharge curves after the initial activation, while the Li^+^ diffusion coefficient (D_Li+_) in the C‐BP electrolyte is slightly higher than that in the control electrolyte. Compared to the C‐BP electrolyte, the polarization in the charge/discharge curves of the cycled LRLO in the control electrolyte becomes severe after 100 cycles, which simultaneously leads to the decreased D_Li+_ value (Figure 3b). These results illustrate that slow electrochemical reaction kinetics exist in the LRLO cathode cycled in the control electrolyte, caused by side‐product accumulation and structural degradation. The stable and superior interfacial kinetics for Li^+^ transportation can be maintained in the LRLO cathode with C‐BP electrolyte due to the effectiveness of the robust CEI film in inhibiting electrolyte decomposition.

Furthermore, the cycling performance in high temperatures serves as another important criterion for evaluating the electrochemical and chemical stability of the electrolyte. Before the high‐temperature test, the half‐cells with different electrolytes were pre‐cycled for three charge/discharge cycles at 30 °C to construct a stable CEI film on the LRLO surface. In Figure 3d, a high discharge capacity of 309.9 mAh g^−1^ at 0.1C can be delivered from the LRLO cathodes with the C‐BP electrolyte at a temperature of 45 °C, while a capacity loss (5.2 mAh g^−1^) occurs in the LRLO cathodes with the control electrolyte. Moreover, as depicted in Figure 3c, the C‐BP electrolyte enables long‐term electrochemical stability of the LRLO cathode, achieving a capacity retention of 86.6% and an average coulombic efficiency (CE) of 99.3% after 400 cycles at 1C. In contrast, the control electrolyte exhibits a significantly lower capacity retention of 49.5% after 150 cycles. The C‐BP electrolyte still allows the LRLO cathode to operate at an elevated temperature of 60 °C, whereas a voltage drop occurs in the Li//LRLO cell with the control electrolyte (Figure S16).

To investigate the chemical properties of the CEI films on the cathodes at high temperature, self‐discharge measurements of LRLO cathodes were evaluated through monitoring voltage drops and residual capacity (Figure 3e). Before the high‐temperature storage, the LRLO cathodes were fully charged to 4.8 V at 0.1C. After storage for three days at 45 °C, the constant‐current discharge was performed on the cells to obtain the capacity loss. Remarkably, the LRLO cathode in the C‐BP electrolyte could maintain a high voltage state (4.09 V) after storage for three days at 45 °C, while the voltage of the LRLO cathode in the control electrolyte rapidly decreased to 3.87 V (inset graph in Figure 3e). Simultaneously, the cell with the C‐BP electrolyte exhibits a smaller capacity loss of 65 mAh g^−1^ than that in the control electrolyte (96 mAh g^−1^). These findings further show that the additive‐derived CEI film on the LRLO surface exhibits superior stability and durability to alleviate the side reaction during extreme conditions.

In addition, the practical application of the C‐BP electrolyte was further validated in the Li//LRLO cells with high loading LRLO and limited lithium, as well as in the graphite//LRLO pouch cell. The stability of Li//Li symmetric cells at the current density of 1 mA cm^−2^ with deposition capacities of 1 and 2 mAh cm^−2^ were first evaluated to determine the compatibility between Li metal and electrolytes. Notably, the C‐BP electrolyte enables stable performance of Li plating/stripping for over1000 and 600 h at the deposition capacity of 1 and 2 mAh cm^−2^, respectively (Figure S17). When the C‐BP electrolyte was tested with a commercial LRLO cathode with a high loading of 15 mg cm^−2^ and 50 µm lithium foil, the Li//LRLO cell with a negative/positive capacity ratio (N/P) of 2.2 shows a 94.4% capacity retention after 200 cycles at the asymmetric 0.2C charge/0.5C discharge protocol (Figure 3g). A rapid capacity fade in the cell using the control electrolyte illustrates incompatibility between the LRLO cathode and carbonate electrolyte without any modification. Figure 3f presents a comparison of the cycling performance of cells with LRLO cycled above 4.55 V, highlighting both our results and those from literature reports. Detailed information can be found in Table S1. The designed C‐BP electrolyte demonstrates excellent capacity retention for the LRLO cathodes, even at both low (4 mg cm^−2^) and high (15 mg cm^−2^) mass loading levels, when cycled at 4.8 V. The half cells with 12 mg cm^−2^ NCM811 cathodes also exhibit good cycling stability in C‐BP electrolyte than control electrolyte at a cut‐off voltage of 4.6 V (Figure S18). It's worth noting that the control electrolyte enables relative electrochemical stability of the NCM811 cathode without ARRs, indicating the ARRs make a notorious effect on electrolyte decomposition in the LRLO cathode. The C‐BP electrolyte delivers superior compatibility with high‐voltage cathodes. In Figure 3h, a practical pouch cell of graphite//LRLO, with a capacity of around 4.5 Ah, was applied to evaluate the universality of the C‐BP electrolyte. The pouch cell, with electrolyte amount/capacity ratio (E/C) of 1.8 g Ah^−1^, weights 57.5 g and delivers 4.5 Ah at 0.1C within the voltage range of 2–4.6 V, thereby achieving a specific energy density of 282.5 Wh kg^−1^ (Figure S19). The electrochemical performance of the pouch cell with the C‐BP electrolyte exhibits extraordinary capacity retention of 83.3% over 50 cycles at 0.2C charge/0.5C discharge. However, the graphite//LRLO pouch cell with the control electrolyte shows severe oxygen release, resulting in capacity loss and cell expansion (Figure S20).

### 2.3 Interfacial Component and Structural Evolution of the LRLO Cathodes

**FIGURE 4:**
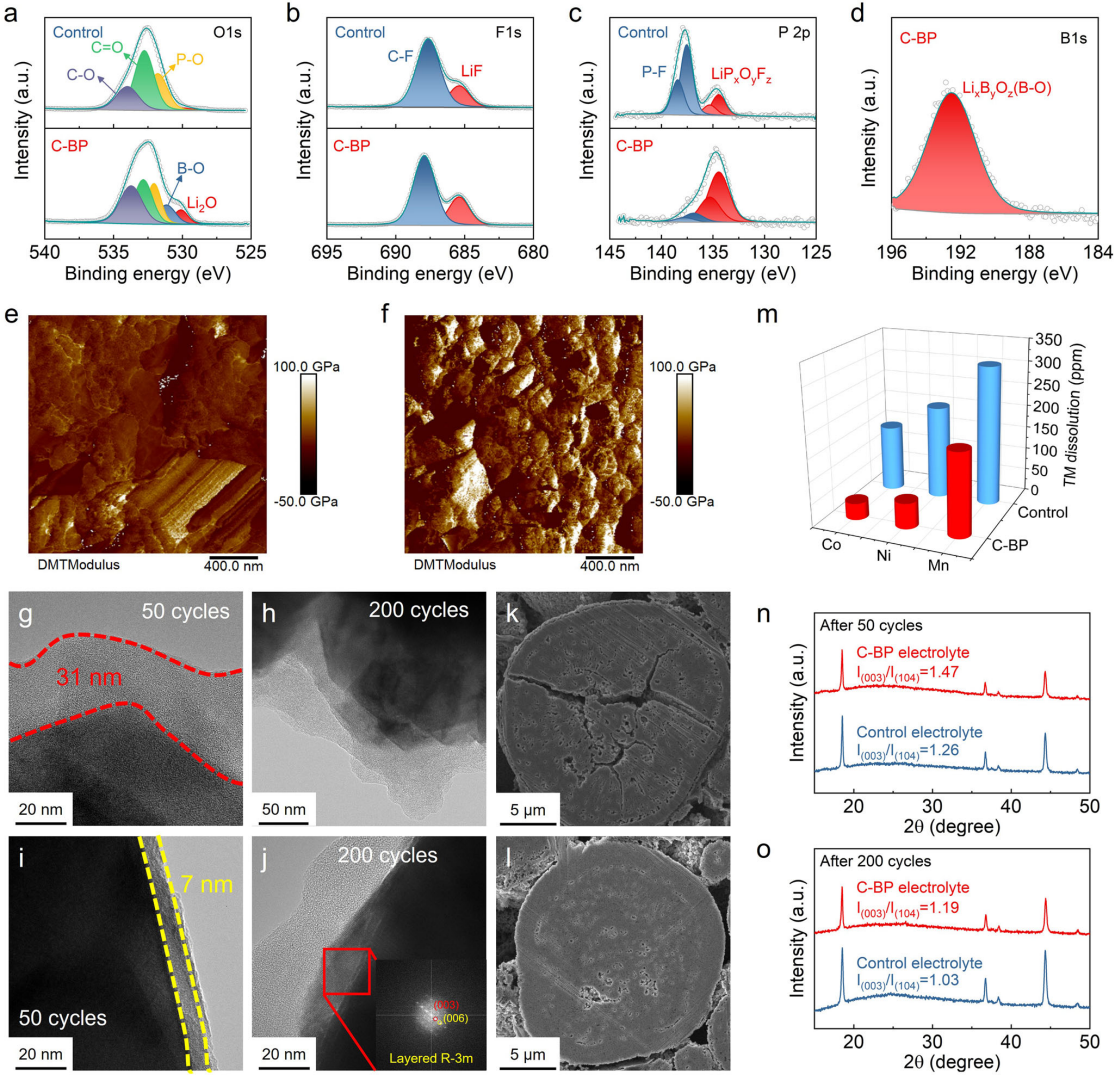
XPS spectra of (a) O 1s, (b) F 1s, (c) P 2p, and (d) B 1s of the LRLO cathodes cycled in the control and C‐BP electrolytes for 50 cycles. DMT modulus obtained from AFM tests of the LRLO cathodes cycled in the (e) control and (f) C‐BP electrolytes. HR‐TEM images of LRLO particles in the (g), (h) control and (i), (j) C‐BP electrolytes after 50 cycles and 200 cycles. Cross‐sectional SEM images of the LRLO particle cycled in (k) control and (l) C‐BP electrolytes after 200 cycles. (m) ICP‐AES measurements of transition metals in the lithium metal anodes obtained from the disassembled Li//LRLO cells after 200 cycles. XRD patterns of the LRLO cathodes using the control and C‐BP electrolytes after (n) 50 and (o) 200 cycles at 1 C current density.

The chemical component of the CEI film on the LRLO surface was further determined by the XPS technique after 50 cycles. The C 1s XPS spectra of the LRLO cathodes with control and C‐BP electrolyte show similar C‐containing species with C‐C, C‐O, and C ═ O, which are related to ROCO_2_Li, ROLi, and Li_2_CO_3_ (Figure S21). According to other XPS spectra in Figure 4a–d, the LiBOB and LiDFOP additives generate a CEI film on the LRLO cathode, enriched with LiP_x_O_y_F_z_, Li_x_B_y_O_z_, Li_2_O, and LiF, before the solvent decomposition occurred. It is worth noting that the content of P‐F species on the LRLO cathode in the control electrolyte after increasing 50 cycles, compared to the initial activation process (Figure 4c; Figure S5). This result indicates that a greater amount of LiPF_6_ undergoes a hydrolysis reaction triggered by solvent decomposition. In contrast, the high content of LiF and LiP_x_O_y_F_z_ on the LRLO cathode in C‐BP electrolyte validates the oxygen scavenge of LiDFOP additives, which suppresses the solvent decomposition. The mechanical strength of CEI of the LRLO cathodes was further evaluated by Derjaguin‐Muller‐Toporov (DMT) modulus obtained from atomic force microscope (AFM) measurement. A high mechanical strength of CEI film is crucial for ensuring the long‐term stability of the LRLO cathodes. After CEI film formation, the average DMT modulus in the C‐BP electrolyte is 22.7 GPa, which is higher than that in the control electrolyte (18.8 GPa), indicating that the CEI film derived from the C‐BP electrolyte provides the merit of high mechanical stability during electrochemical cycling (Figure S22).

The morphology evolution of the cycled LRLO cathodes was investigated by a high‐resolution transmission electron microscope (HR‐TEM) and a scanning electron microscope (SEM). As shown in Figure 4g,i, the surface of the primary LRLO particle is covered with a non‐uniform CEI film approximately 31 nm thick, whereas the C‐BP electrolyte generates a thin and robust CEI film with a thickness of about 7 nm. After 200 cycles, the morphology of the CEI film from the control electrolyte becomes more irregular compared to that formed from the C‐BP electrolyte (Figure 4h,j). The structural evolution from layered to spinel structure in the LRLO particle with control electrolyte indicates severe electrolyte decomposition and interfacial instability (Figure S23). However, the layered structure can be well‐preserved on the surface of the LRLO particle cycled in C‐BP electrolyte, suggesting the formed CEI film contributes to maintaining the structural integrity and enhancing the reversibility of anionic oxygen reaction (Figure 4j). As shown in Figure 4k and Figure S24a, significant cracks and numerous holes are evident in the inner LRLO particles cycled in the control electrolyte, highlighting considerable oxygen loss and substantial strain. In contrast, the LRLO particles cycled in C‐BP electrolyte exhibit excellent structural integrity, characterized by fewer holes and an absence of cracks (Figure 4l; Figure S24b).

TMs dissolution from the cathode to the electrolyte is a notorious and common problem among layered transition metal oxide materials. The inhibition of TMs dissolution in the electrolyte contributed to stabilizing the lattice structure and maintaining a high reversible capacity during cycling. The dissolution of TMs of the LRLO cathode could be determined by measuring the concentration of TMs reduced on the lithium anode surface. As depicted in Figure 4m, the concentrations of Co, Ni, and Mn in the lithium anode disassembled from Li//LRLO cells cycled in the control electrolyte for 200 cycles are 147, 206, and 309 ppm, respectively. In contrast, after cycling in the C‐BP electrolyte, the concentrations of Co, Ni, and Mn are reduced to 37, 57, and 185 ppm, respectively. Furthermore, energy dispersive spectroscopy (EDS) mapping is conducted to evaluate the element distribution from the surface to the bulk structure of LRLO cathodes. The concentrations of O, Mn, Ni, and Co elements in the surface layer of the LRLO particles gradually diminish with increased depth, indicating the LRLO particles suffer from significant dissolution of TMs and the release of oxygen throughout the cycling process in the control electrolyte (Figure S25a,b). In contrast, a narrow depletion region measuring 15 nm in thickness is observed in the LRLO particles cycled in C‐BP electrolyte, which demonstrates a reduced loss of active material (Figure S25c,d). This suggests that the interfacial film formed from the C‐BP electrolyte serves as a more effective barrier against TMs dissolution compared to the control electrolyte, thereby helping to suppress structural deterioration and reduce the loss of active material.

The structural evolution during cycling was detected by the X‐ray diffraction (XRD) of LRLO cathodes with different electrolytes before and after long‐term cycling. In typical layered transition metal oxide cathodes, a higher intensity ratio of (003) and (104) diffraction peaks reflects a smaller degree of cation mixing and better layered structure retention. After 50 cycles, the intensity ratio of (003)/(104) for the cycled LRLO cathodes in the C‐BP electrolyte decreases slightly to 1.47, which remains significantly higher than that of 1.26 for the control electrolyte (Figure 4n). When the charge/discharge cycle reaches 200, the LRLO cathode using the control electrolyte suffers from severe structural degradation, which is linked to the low I(003)/I(104) ratio of 1.03 (Figure 4o). The variation in the valence state of the Mn elements indicates the structural stability of LRLO cathodes, which significantly influences the electrochemical performance of the cathode. In Figures S26 and S27, XPS analysis was conducted to assess the valence states of the Mn element in the LRLO cathodes following 200 cycles. The energy difference between the multiple peaks of the Mn 3s splitting (*ΔE_3s_*) is regarded as a reliable indicator of the valence state of Mn. After 200 cycles, the *ΔE_3s_* for the LRLO cathode tested in the C‐BP electrolyte varied from 4.43 to 6.06 eV, whereas the *ΔE_3s_* of the LRLO cathode cycled in the control electrolyte increased to 6.77 eV. The proportions of Mn^4+^ in the LRLO cathodes cycled in the control and C‐BP electrolytes decrease from 85.7% to 29.4% and 67.9%, respectively, suggesting that the Jahn–Teller effect induced by Mn^3+^ is significantly suppressed when cycled in the C‐BP electrolyte. The Raman spectroscopy of the cycled LRLO cathodes reflects the structural change from layered to spinel structure. The peak at around 600 cm^−1^ corresponds to the stretching A_1g_ mode, which belongs to the layered R‐3m structure (Figure S28). The peak of spinel structure is located at 650 cm^−1^. After 200 cycles, an intensified signal of spinel structure in the LRLO cathode indicates the severe structural evolution when cycling in the control electrolyte, while the layered structure can be well maintained in the LRLO cathode cycled in the C‐BP electrolyte.

### 2.4 Mechanism of the Stable CEI Formation and Enhanced Electrochemical Performance

**FIGURE 5:**
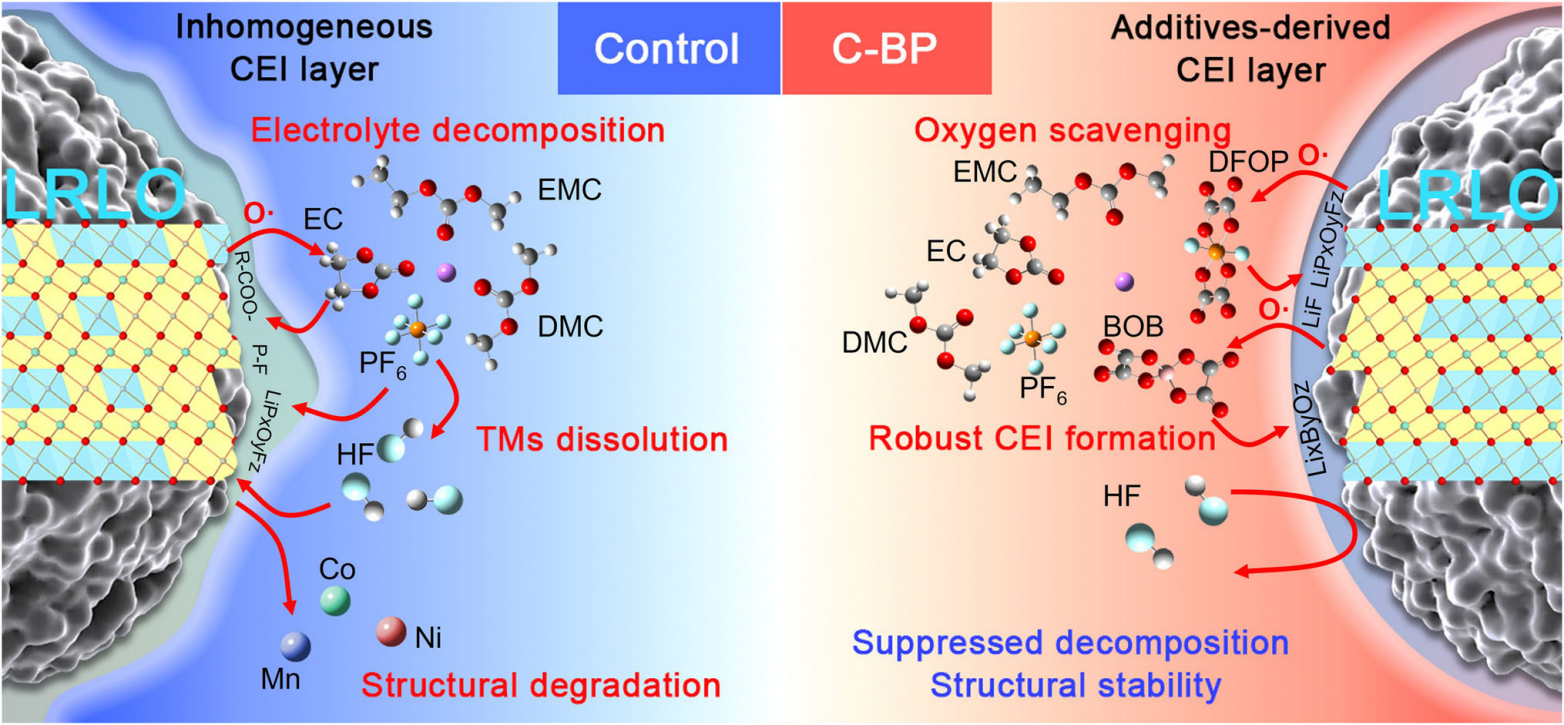
Schematic diagram of the mechanism of oxygen scavenging and CEI formation for improving the electrochemical performance of LRLO cathodes.

The mechanism of electrolyte decomposition and structural degradation of the LRLO cathode is summarized in Figure 5. Simultaneously, the effects of LiBOB and LiDFOP additives on CEI formation and oxygen scavenging are further reveal, contributing to the enhanced electrochemical performance of the LRLO cathode. In the control electrolyte, the carbonate component is subjected to nucleophilic attack by active oxygen species released from the LRLO cathode at high voltage [45]. Furthermore, the side products trigger the hydrolysis of LiPF_6_, which produces HF acid species. A severe corrosion of the LRLO cathode by HF renders the TMs dissolution. Simultaneously, the inhomogeneous CEI film derived from the control electrolyte can not inhibit the above‐mentioned side reaction. During progressive cycling, the irreversible lattice oxygen release and TMs dissolution accelerate structural degradation of LRLO, which renders capacity loss and electrolyte consumption. In contrast, LiBOB and LiDFOP additives with electron‐deficient B/P centers act as oxygen scavengers to construct a robust and homogeneous CEI film on the LRLO surface. First, the addition of LiBOB and LiDFOP enhances the reversibility of anionic redox reaction and inhibits oxygen species release. Second, the CEI film derived from additives isolates the direct contact between electrolyte and LRLO cathode, which suppresses electrolyte decomposition caused by nucleophilic attack. Third, the suppressed LiPF_6_ hydrolysis decreases the production of harmful HF species, which benefits the structural stability and enhances electrochemical performance. Thereby, the LRLO cathode using the C‐BP electrolyte exhibits long‐term cyclic stability with excellent capacity retention.

## 3 Conclusions

The nucleophilic attack from reactive oxygen species is a main reason for the incompatibility between the carbonate electrolyte and the high‐voltage LRLO cathode. In this work, we developed a novel electrolyte featuring oxygen scavenging additives for a high‐voltage LRLO cathode with high mass loading, achieving a high capacity retention after long‐term performance. The exceptional performance is attributed to the scavenging of oxygen species, which leads to a robust CEI film formation. The formed CEI film effectively enhance reversibility of anionic redox reaction and inhibits electrolyte decomposition caused by nucleophilic attack. Therefore, the LRLO cathode in C‐BP electrolyte exhibits structural stability with reduced TMs dissolution and a decrease in lattice oxygen release. Exceptional cyclic stability is achieved in the high mass loading (15 mg cm^−2^) LRLO cathode in the C‐BP electrolyte, demonstrating a high capacity retention of 94.4% after 200 cycles. The applicability of C‐BP electrolyte is demonstrated through the cyclic performance of a 4.5 Ah graphite//LRLO pouch cell, achieving a high energy density of 282.5 Wh kg^−1^. This work lays the groundwork for using electrolyte engineering to optimize the LRLO cathode with a reversible anionic redox reaction and supports the design of electrode–electrolyte interfaces for high‐energy‐density LIBs with long‐term stability.

## Author Contributions

J. C., H. M., J. H. conceived the idea, conducted the experiments, performed the formal analyses, and wrote the manuscript; H. W., Z. Y. conducted the experiment and performed the formal analyses; C. L., Z. W. performed the formal analyses and revised the manuscript; J. Z., M. Z., J. L. provided funding support and revised the manuscript. All authors commented on the manuscript.

## Conflicts of Interest

The authors declare no conflicts of interest.

## Supporting information




**Supporting File**: advs73984‐sup‐0001‐SuppMat.docx.

## Data Availability

The data that support the findings of this study are available in the supplementary material of this article.

## References

[1] C.‐Y. Wang , T. Liu , X.‐G. Yang , et al., “Fast Charging of Energy‐Dense Lithium‐Ion Batteries,” Nature 611 (2022): 485–490. 36224388 10.1038/s41586-022-05281-0

[2] J. T. Frith , M. J. Lacey , and U. Ulissi , “A Non‐Academic Perspective on the Future of Lithium‐Based Batteries,” Nature Communications 14 (2023): 420. 10.1038/s41467-023-35933-2 PMC9879955 36702830

[3] J. Zhou , X. Zhou , W. Yu , Z. Shang , and S. Xu , “Towards Greener Recycling: Direct Repair of Cathode Materials in Spent Lithium‐Ion Batteries,” Electrochemical Energy Reviews 7 (2024): 13.

[4] L. Sun , Y. Liu , L. Wang , and Z. Jin , “Advances and Future Prospects of Micro‐Silicon Anodes for High‐Energy‐Density Lithium‐Ion Batteries: a Comprehensive Review,” Advanced Functional Materials 34 (2024): 2403032.

[5] F. Schomburg , B. Heidrich , S. Wennemar , et al., “Lithium‐Ion Battery Cell Formation: Status and Future Directions towards a Knowledge‐Based Process Design,” Energy & Environmental Science 17 (2024): 2686–2733.

[6] Y. Fan , W. Zhang , Y. Zhao , Z. Guo , and Q. Cai , “Fundamental Understanding and Practical Challenges of Lithium‐Rich Oxide Cathode Materials: Layered and Disordered‐Rocksalt Structure,” Energy Storage Materials 40 (2021): 51–71.

[7] J. Lu , C. Xu , W. Dose , et al., “Microstructures of Layered Ni‐Rich Cathodes for Lithium‐Ion Batteries,” Chemical Society Reviews 53 (2024): 4707–4740. 38536022 10.1039/d3cs00741c

[8] A. Zhang , Z. Bi , G. Wang , et al., “Regulating Electrode/Electrolyte Interfacial Chemistry Enables 4.6 V Ultra‐Stable Fast Charging of Commercial LiCoO_2_ ,” Energy & Environmental Science 17 (2024): 3021–3031.

[9] B. Cui , Z. Xiao , S. Cui , S. Liu , X. Gao , and G. Li , “Safety Issues and Improvement Measures of Ni‐Rich Layered Oxide Cathode Materials for Li‐Ion Batteries,” Electrochemical Energy Reviews 7 (2024): 27.

[10] J. Xiang , Y. Wei , Y. Zhong , et al., “Building Practical High‐Voltage Cathode Materials for Lithium‐Ion Batteries,” Advanced Materials 34 (2022): 2200912. 10.1002/adma.202200912 35332962

[11] H. Wan , J. Xu , and C. Wang , “Designing Electrolytes and Interphases for High‐Energy Lithium Batteries,” Nature Reviews Chemistry 8 (2023): 30–44. 38097662 10.1038/s41570-023-00557-z

[12] W. Zuo , M. Luo , X. Liu , et al., “Li‐Rich Cathodes for Rechargeable Li‐Based Batteries: Reaction Mechanisms and Advanced Characterization Techniques,” Energy & Environmental Science 13 (2020): 4450–4497.

[13] Q. Huang , J. Liu , X. Chen , et al., “Recent Progress and Challenges of Li‐Rich Mn‐Based Cathode Materials for Solid‐State Lithium‐Ion Batteries,” Advanced Materials 37 (2025): 2410006. 10.1002/adma.202410006 39686794

[14] Y. Lei , J. Ni , Z. Hu , et al., “Surface Modification of Li‐Rich Mn‐Based Layered Oxide Cathodes: Challenges, Materials, Methods, and Characterization,” Advanced Energy Materials 10 (2020): 2002506.

[15] X. Yuan , T. Dong , J. Liu , et al., “Bi‐Affinity Electrolyte Optimizing High‐Voltage Lithium‐Rich Manganese Oxide Battery via Interface Modulation Strategy,” Angewandte Chemie International Edition 62 (2023): 202304121. 10.1002/anie.202304121 37226711

[16] J. Song , H. Wang , Y. Zuo , et al., “Building Better Full Manganese‐Based Cathode Materials for Next‐Generation Lithium‐Ion Batteries,” Electrochemical Energy Reviews 6 (2023): 20.

[17] X. Ji , Q. Xia , Y. Xu , H. Feng , P. Wang , and Q. Tan , “A Review on Progress of Lithium‐Rich Manganese‐Based Cathodes for Lithium Ion Batteries,” Journal of Power Sources 487 (2021): 229362.

[18] K. Luo , M. R. Roberts , R. Hao , et al., “Charge‐Compensation in 3D‐Transition‐Metal‐Oxide Intercalation Cathodes through the Generation of Localized Electron Holes on Oxygen,” Nature Chemistry 8 (2016): 684–691. 10.1038/nchem.2471 27325095

[19] W. E. Gent , K. Lim , Y. Liang , et al., “Coupling between Oxygen Redox and Cation Migration Explains Unusual Electrochemistry in Lithium‐Rich Layered Oxides,” Nature Communications 8 (2017): 2091. 10.1038/s41467-017-02041-x PMC5727078 29233965

[20] T. Peng , Y. Zhao , Q. Liu , et al., “Surface and Interfacial Modulation of Lithium‐Rich Manganese Layered Oxide Cathode Materials: Progress and Challenges,” Small 21 (2025): 2412236. 10.1002/smll.202412236 40151015

[21] W. Kong , C. Zhao , S. Sun , et al., “From Liquid to Solid‐State Batteries: Li‐Rich Mn‐Based Layered Oxides as Emerging Cathodes with High Energy Density,” Advanced Materials 36 (2024): 2310738. 10.1002/adma.202310738 38054396

[22] M. Yang , T. Chen , G. Wang , et al., “A Surface‐To‐Interface Boronation Engineering Strategy Stabilizing the O/Mn Redox Chemistry of Lithium‐Rich Manganese Based Oxides towards High Energy‐Density Cathodes,” Energy & Environmental Science 18 (2025): 6168–6179.

[23] R. A. House , J.‐J. Marie , M. A. Pérez‐Osorio , G. J. Rees , E. Boivin , and P. G. Bruce , “The Role of O_2_ in O‐Redox Cathodes for Li‐Ion Batteries,” Nature Energy 6 (2021): 781–789.

[24] X. Li , Y. Qiao , S. Guo , et al., “Direct Visualization of the Reversible O^2−^/O^−^ Redox Process in Li‐Rich Cathode Materials,” Advanced Materials 30 (2018): 1705197. 10.1002/adma.201705197 29457283

[25] B. Zhang , X. Wu , H. Luo , et al., “Gradient Interphase Engineering Enabled by Anionic Redox for High‐Voltage and Long‐Life Li‐Ion Batteries,” Journal of the American Chemical Society 146 (2024): 4557–4569. 38345667 10.1021/jacs.3c11440

[26] E. Castel , E. J. Berg , M. El Kazzi , P. Novák , and C. Villevieille , “Differential Electrochemical Mass Spectrometry Study of the Interface of X Li_2_MnO_3_ (1– x )LiMO_2_ (M = Ni, Co, and Mn) Material as a Positive Electrode in Li‐Ion Batteries,” Chemistry of Materials 26 (2014): 5051–5057.

[27] F. Li , Y. Lin , J. Liu , et al., “Boosting Oxygen Redox Reversibility in Chemo‐Mechanically Robust Li‐Rich Oxide Cathodes via Multi‐Scale Defect Design,” Energy & Environmental Science 18 (2025): 1241–1254.

[28] G. Lim , M. K. Cho , J. Choi , et al., “Decoupling Capacity Fade and Voltage Decay of Li‐Rich Mn‐Rich Cathodes by Tailoring Surface Reconstruction Pathways,” Energy & Environmental Science 17 (2024): 9623–9634.

[29] Q. Zhang , S. Xu , H. Zhu , et al., “Synchronous Dual Additives To Boost Multiphase Interface Stability of High‐Voltage Li‐Rich Mn‐Based Batteries,” Journal of Materials Chemistry A 13 (2025): 399–408.

[30] Y. Liu , C. Zhao , J. Du , X. Zhang , A. Chen , and Q. Zhang , “Research Progresses of Liquid Electrolytes in Lithium‐Ion Batteries,” Small 19 (2023): 2205315. 10.1002/smll.202205315 36470676

[31] X. Liu , Y. Li , J. Liu , H. Wang , X. Zhuang , and J. Ma , “570 Wh Kg^−^ ^1^‐Grade Lithium Metal Pouch Cell with 4.9V Highly Li^+^ Conductive Armor‐like Cathode Electrolyte Interphase via Partially Fluorinated Electrolyte Engineering,” Advanced Materials 36 (2024): 2401505. 10.1002/adma.202401505 38437452

[32] C. Zhang , Z. Lu , M. Song , et al., “Highly Oxidation‐Resistant Ether Gel Electrolytes for 4.7 V High‐Safety Lithium Metal Batteries,” Advanced Energy Materials 13 (2023): 2203870.

[33] X. Lan , S. Yang , T. Meng , C. Zhang , and X. Hu , “A Multifunctional Electrolyte Additive with Solvation Structure Regulation and Electrode/Electrolyte Interface Manipulation Enabling High‐Performance Li‐Ion Batteries in Wide Temperature Range,” Advanced Energy Materials 13 (2023): 2203449.

[34] S. Tan , Z. Shadike , J. Li , et al., “Additive Engineering for Robust Interphases To Stabilize High‐Ni Layered Structures at Ultra‐High Voltage of 4.8 V,” Nature Energy 7 (2022): 484–494.

[35] G. Kang , G. Zhong , K. Cai , et al., “Dimethyl Sulfide Electrolyte Additive Enabled High‐Voltage Lithium‐Ion Battery,” ACS Energy Letters 9 (2024): 2572–2581.

[36] J.‐G. Han , J. B. Lee , A. Cha , et al., “Unsymmetrical Fluorinated Malonatoborate as an Amphoteric Additive for High‐Energy‐Density Lithium‐Ion Batteries,” Energy & Environmental Science 11 (2018): 1552–1562.

[37] B. Zhang , L. Wang , X. Wang , et al., “Sustained Releasing Superoxo Scavenger for Tailoring the Electrode‐Electrolyte Interface on Li‐Rich Cathode,” Energy Storage Materials 53 (2022): 492–504.

[38] J. Li , J. Yang , Z. Ji , et al., “Prospective Application, Mechanism, and Deficiency of Lithium Bis(oxalate)Borate as the Electrolyte Additive for Lithium‐Batteries,” Advanced Energy Materials 13 (2023): 2301422.

[39] A. Zhang , Z. Bi , E. Yang , et al., “Formulating Electrophilic Electrolyte for in Situ Stabilization of 4.8 V Li‐Rich Batteries with 100% Initial Coulombic Efficiency,” Angewandte Chemie International Edition 64 (2025): 202502603. 10.1002/anie.202502603 40051192

[40] Z. Liu , Z. Liu , K. Li , et al., “Exploring Trimethyl‐Phosphate‐Based Electrolytes without a Carbonyl Group for Li‐Rich Layered Oxide Positive Electrodes in Lithium‐Ion Batteries,” The Journal of Physical Chemistry Letters 13 (2022): 11307–11316. 36453838 10.1021/acs.jpclett.2c02585

[41] Z. Lu , D. Liu , K. Dai , et al., “Tailoring Solvation Chemistry in Carbonate Electrolytes for all‐Climate, High‐Voltage Lithium‐Rich Batteries,” Energy Storage Materials 57 (2023): 316–325.

[42] J. Liu , J. Wang , Y. Ni , K. Zhang , F. Cheng , and J. Chen , “Recent Breakthroughs and Perspectives of High‐Energy Layered Oxide Cathode Materials for Lithium Ion Batteries,” Materials Today 43 (2021): 132–165.

[43] Z. Wang , Y. Wang , B. Py , et al., “Freely Accessible Distribution of Relaxation Times Analysis for Electrochemical Impedance Spectroscopy,” ACS Electrochemistry 1 (2025): 2680–2689.

[44] Y. Lu , C.‐Z. Zhao , J.‐Q. Huang , and Q. Zhang , “The Timescale Identification Decoupling Complicated Kinetic Processes in Lithium Batteries,” Joule 6 (2022): 1172–1198.

[45] J. Huang , H. Zhang , X. Yuan , et al., “Regulating Robust Interphase Using a Functional Ionic Liquid Additive with Bi‐Electrode Affinity To Stabilize the High‐Voltage Lithium‐Rich Lithium Metals Batteries,” Chemical Engineering Journal 464 (2023): 142578.

