# Supplementary material for: Interface Construction via Scavenging Oxygen Species Enables High‐Performance Li‐Rich Layered Oxide Cathode
Source: Adv Sci (Weinh). 2026 Jan 21;13(18):e24384. doi: 10.1002/advs.202524384 (PMC13042874; doi:10.1002/advs.202524384)
Supplement: Supplementary file 1 — Supporting File: advs73984‐sup‐0001‐SuppMat.docx. [file ADVS-13-e24384-s001.docx]

**Supporting Information**

**Interface construction via scavenging oxygen species enables high-performance Li-rich layered oxide cathode**

Jiahe Chen^1,†^, Haoran Ma^1,†^, Jiajia Huang^1,†^, Hongbo Wu^1^, Zhen Yang^1^, Chenchen Li^2^, Zhijun Wu^2^, Jingwei Zhao^3,^*, Min Zhu^1^, Jun Liu^1,^*

^1^Guangdong Provincial Key Laboratory of Advanced Energy Storage Materials, School of Materials Science and Engineering, South China University of Technology, Guangzhou, 510641, China.

^2^Institute of Science and Technology for New Energy, Xi’an Technological University, Xi’an, 710021, China.

^3^Guangzhou Tinci Materials Technology Co. Ltd., Guangzhou, 510700, China.

*To whom correspondence should be addressed: [msjliu@scut.edu.cn](mailto:msjliu@scut.edu.cn), zhaojingwei@tinci.com

^†^These authors contributed equally to this work.

**Experimental Details**

**1. Electrolytes and Electrodes Preparation**

Lithium hexafluorophosphate (LiPF_6_), lithium bis(oxalate)borate (LiBOB), ethylene carbonate (EC), dimethyl carbonate (DMC), ethyl methyl carbonate (EMC) were purchased from DodoChem Co., Ltd. Lithium difluorobis(oxalato)phosphate (LiDFOP) was provided by Macklin Inc. The solvents were dried by 4 Å molecular sieves before utilization. All electrolyte preparations were conducted in an argon filled glove box with water and oxygen content below 0.1 ppm. Typically, the control electrolyte was prepared by dissolving 1.2 M LiPF_6_ into mixed solvents of EC, DMC, and EMC with a volume ratio of 1:1:1. The C-BP electrolyte was prepared by adding 0.1 M concentrations of LiBOB and LiDFOP additives into the carbonate electrolyte (1 M LiPF_6_ in EC:DMC:EMC with a volume ratio of 1:1:1).

The LRLO (Li_1.12_Mn_0.49_Ni_0.16_Co_0.16_O_2_) cathode material was provided by Youyan Technology Co., Ltd. without any treatment. KS-6 and Super-P conductive regents, poly(vinylidenefluoride) binder (PVDF), and N-methyl-2-pyrrolidone (NMP) were purchased from Canrd Technology Co. Ltd. The cathode slurry was combined with LRLO material powder, KS-6 conductive regent, Super-P conductive regent, and PVDF binder in an 80:5:5:10 weight ratio, as well as NMP solvent. Then, the slurry was stirred evenly and coated on the Al foil. The obtained electrode was dried at 60 °C to remove residual NMP and then placed at 120 °C for 12 h under a vacuum condition. The mass loading of the cathodes is controlled at around 4 mg cm^-2^. The electrode was sliced into 12-mm-diameter discs for typical electrochemical tests. The 14 mm-diameter Li foil with 400 μm thickness was bought from Canrd Technology Co. Ltd.

The graphite//LRLO pouch cells were fabricated by the winding method. The areal loading of graphite and LRLO are 12.9 and 13.3 mg cm^-2^, respectively. The graphite//LRLO pouch cells were placed at 45 °C for 24 hours after injecting electrolytes. After activation and cell aging, the secondary sealing process was completed in a sealing machine (MSK-115A-LS, Kejing Co. Ltd.).

**2. Electrochemical measurements**

The cycling performance and electrochemical tests of batteries were conducted with 2025-type coin cells, consisting of Li foil, as-prepared electrode, and Celgard 2325 separators soaked with 50 μL electrolyte. The constant-current charge and discharge cycling performances of Li//LRLO half cells were measured on a CT2001A battery test system (Wuhan LAND Electronic Co., Ltd.). The Li//LRLO cells were charged to 4.8 V and discharged to 2.0 V at 0.1 C rate in the initial three cycles for activation. After that, the cells were cycled at 1 C rate for the subsequent cycles. The rate test was set at the half cells corresponding to 0.1C, 0.2C, 0.5C, 1C, 2C, and 5C. Leakage currents of Li//LRLO half cells were measured by holding at different voltages for 24 hours from 4.8 to 5.0 V. The cycling performance of the graphite//LRLO pouch cells were recorded on a multichannel CT-4008Tn-5V6A battery cycler (Neware Co., Ltd.). The oxidation potential was evaluated through the linear sweep voltammetry (LSV) test with Li//Pt cells scanning from the open circuit to 6 V with a scan rate of 0.5 mV s^-1^, performed on Gamry Interface 1000E. Cyclic voltammetry (CV) was conducted to determine interfacial kinetics of LRLO from 2.0-4.8 V at scan rates of 0.2, 0.4, 0.6, 0.8, 1.0, and 1.2 mV s^-1^. Electrochemical impedance spectroscopy (EIS) of cycled electrodes was tested with a frequency range from 0.01 Hz to 1 MHz and an amplitude voltage of 5 mV. The distribution of relaxation time analysis was processed by the Matlab Graphical used DRTtools, which was developed by Ciucci’s research group^[1–3]^.

In addition, Li^+^ transference numbers (*t_Li+_*) of the electrolytes were tested by using potentiostatic polarization technique and electrochemical impedance spectroscopy (EIS) on symmetric Li//Li cells. Polarization voltage (*∆V*=10 mV) was loaded on the symmetric cell for 7200 s. Initial state current (*I_0_*) and stead state current (*I_ss_*) were recorded before and after the polarization process. While EIS data was measured before and after the polarization with the frequency range from 100 mHz to 1 MHz. The transference number was calculated by the following equation:

$t_{Li+}=\frac{I_{ss}(\Delta V-I_{0}R_{0})}{I_{0}(\Delta V-I_{ss}R_{ss})}$ (1)

where R_0_ and R_ss_ is the cell impedance of the initial and steady state, respectively.

Galvanostatic intermittent titration technique (GITT) measurements was conducted to calculate Li^+^ diffusion coefficient (*D_Li+_*) of LRLO at initial activated states and after 100 cycles. The Li//LRLO half cells were charged or discharged at 0.1C with a 10-min current pulse and a 1 h relaxation period in each. The GITT was evaluated at 30 ^o^C by the CT2001A battery test system (Wuhan LAND Electronic Co., Ltd.). The *D_Li+_* can be calculated by the following equation:

$D_{Li+}=\frac{4}{\pi\tau}{(\frac{m_{B}V_{M}}{M_{B}S})}^{2}{(\frac{{\Delta E}_{s}}{{\Delta E}_{t}})}^{2}$ (1)

where *m_B_* is the weight of the active material, *V_M_* is the molar volume of the active material, *M_B_* is the molar mass of the active material, and *S* is the area of the electrode. *∆E_s_* and *∆E_t_* is the change in steady-state voltage and transient voltage, respectively.

The activation energy (*E_a_*) was derived from the EIS data of Li//Li symmetry cells at varied temperatures. The value of *E_a_* was calculated from the Arrhenius equation as follows:

$k=\frac{T}{R_{ct}}=Aexp\left( -\frac{E_{a}}{R} \right)$ (2)

where *k* represents the rate constant, *T* is the absolute temperature, *R_ct_* represents the ion transfer resistance, *A* is the preexponential constant, *E_a_* is the activation energy, and R is the standard gas constant.

**Characterizations**

All the cells were disassembled in an argon-filled glovebox with O_2_ and H_2_O contents below 0.1 ppm. The disassembled electrodes were washed in pure DMC solvent three times to remove the electrolyte, and then placed in the glovebox to evaporate DMC solvent. The surface morphologies of cycled LRLO electrodes were observed by transmission electron microscopy (Thermo Fisher Talos F200x). The structural characterization of cycled LRLO electrodes was carried out by X-ray diffraction (XRD). The chemical composition of cycled LRLO electrodes was analyzed by using X-ray photoelectron spectroscopy (XPS, Thermo Scientific ESCALAB XI+), excited by an Al Kα radiation source. Ion conductivities of as-prepared electrolytes were measured by a conductivity analyzer (Leici DDB-303A) under an argon-filled glovebox after calibration with saturated KCl aqueous solution. Inductively coupled plasma atomic emission spectrometry (ICP-AES, iCAP 7200 Duo, Thermofisher Scientific) was used to measure the transition metal content on the lithium electrodes. Atomic force microscopy (Bruker Dimension Icon) was applied to measure the Young’s modulus of the CEI film of cycled LRLO cathodes in different electrolytes. The EPR spectra were obtained on CIQTEK EPR200-PLUS electron paramagnetic resonance spectrometer. A baseline sample was prepared that contained 0.03 M KO_2_, used as a superoxide radical equivalent, and 0.15 M DMPO, used as a spin-trapping agent to stabilize the superoxide radicals, dissolved in anhydrous acetonitrile. The Li-salts containing samples were prepared by introducing 0.03 M Li salt in the baseline sample. Gas evolution of Li//LRLO cells in different electrolytes was performed using online differential electrochemical mass spectrometry (Pro-tech) at the electrochemical voltage range of 2-4.8 V.

HOMO and LUMO energies were calculated by density function theory. Ground-state geometries were optimized at the B3LYP/6-31G* level^[4,5]^. And then the energy levels of HOMO and LUMO were calculated at the B3LYP/6-311++G(d,p) basis set^[6]^. All the calculations were performed using the Gaussian 16 program^[7]^.


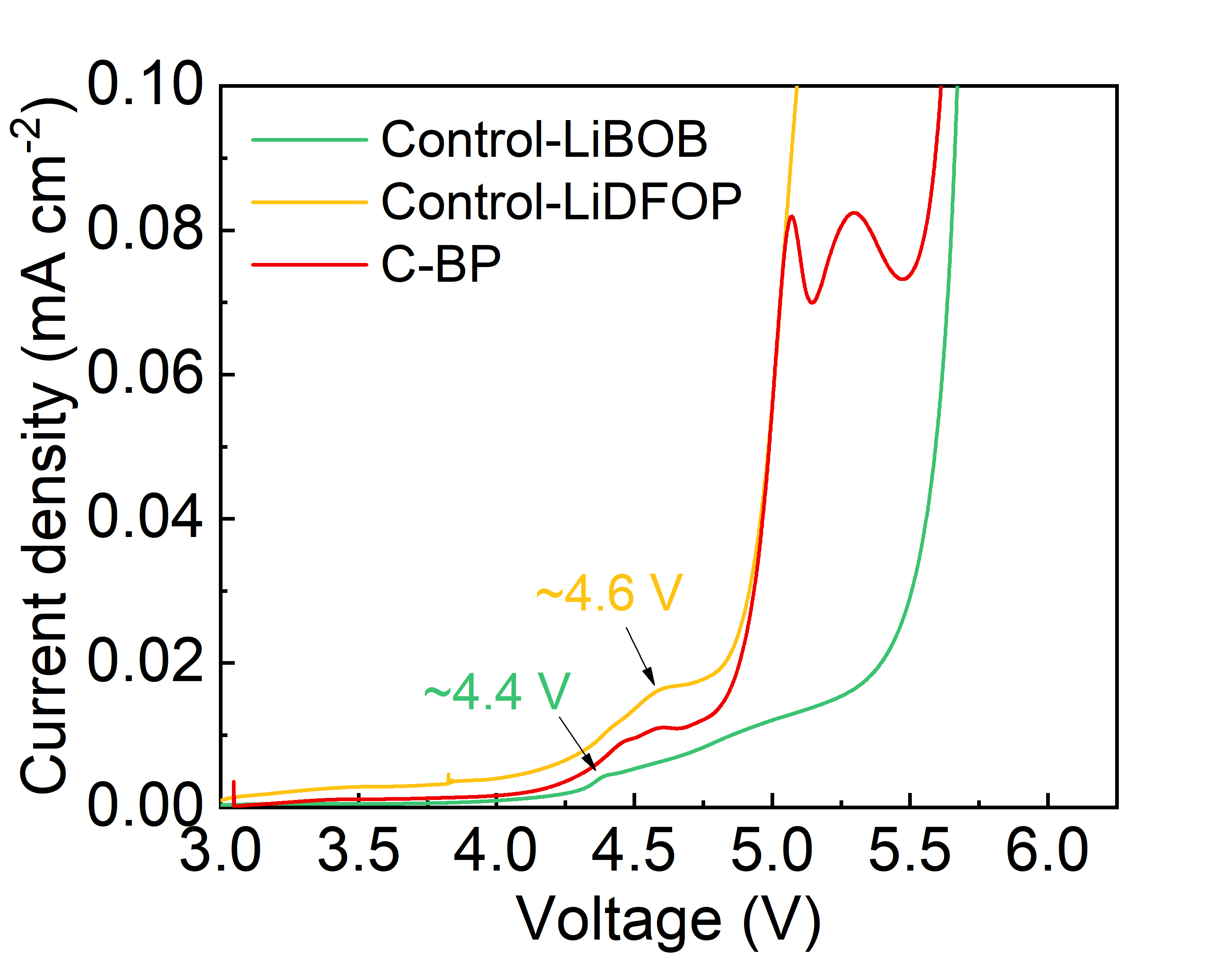


**Figure S1** Linear sweep voltammetry profiles of Li//Pt cells in the control electrolytes with 0.1 M LiBOB (control-LiBOB) or LiDFOP (control-LiDFOP) and C-BP electrolyte at the scanning rate of 0.5 mV s^-1^.


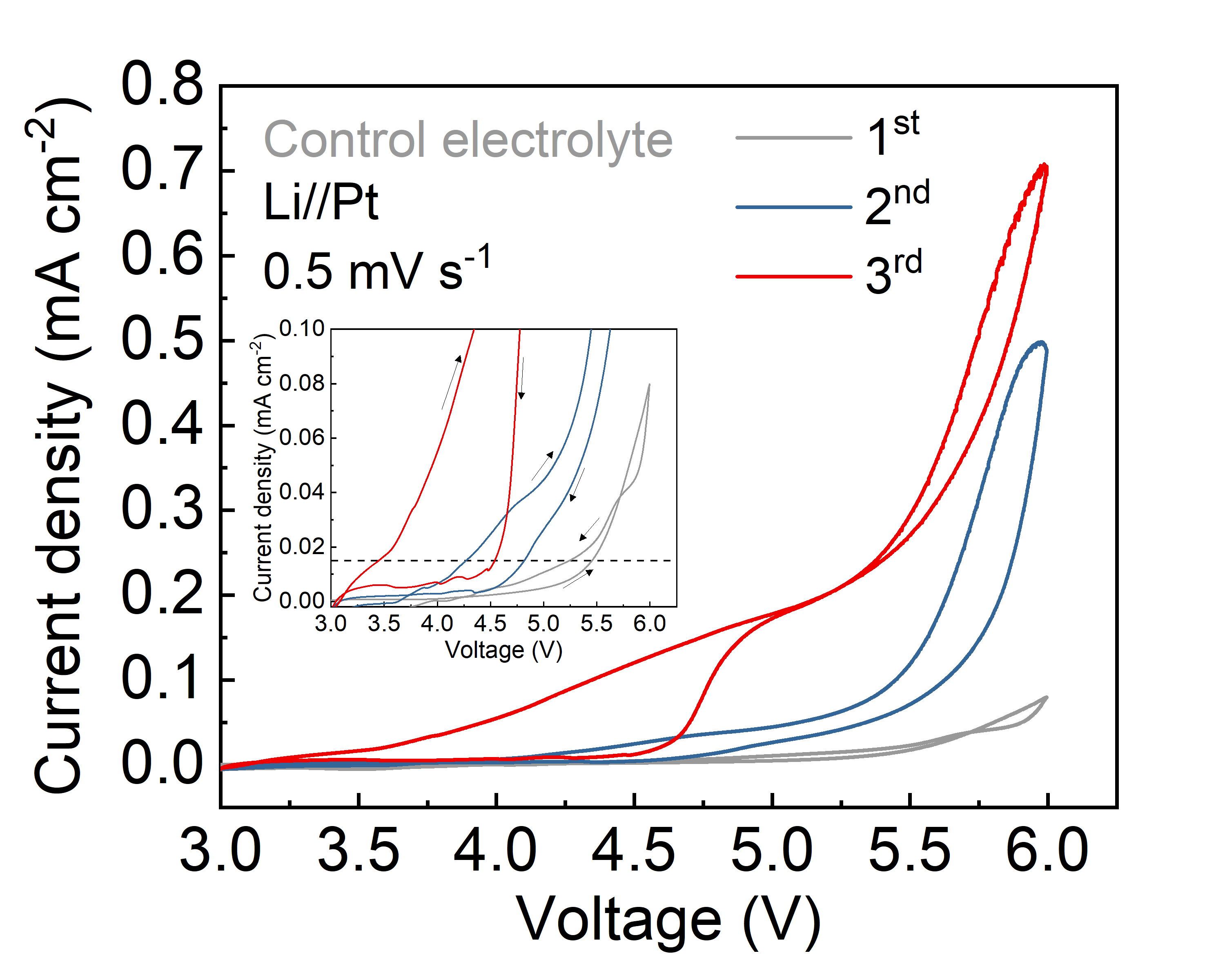


**Figure S2** Cyclic voltammetry profiles of Li//Pt cells with the control electrolyte at the scanning rate of 0.5 mV s^-1^.


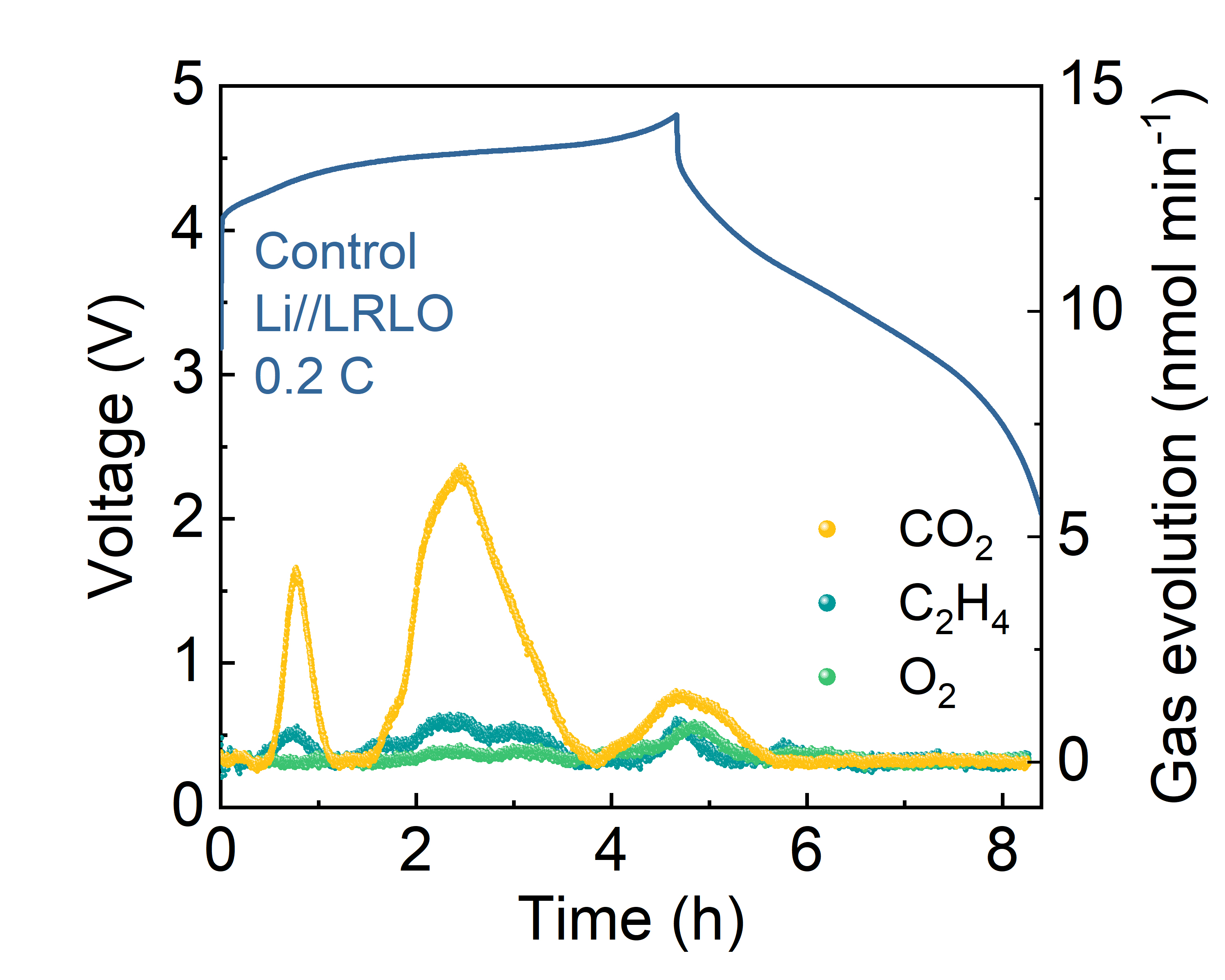


**Figure S3** DEMS gas analysis for Li//LRLO cells in control electrolyte during first charge and discharge at 0.2 C.


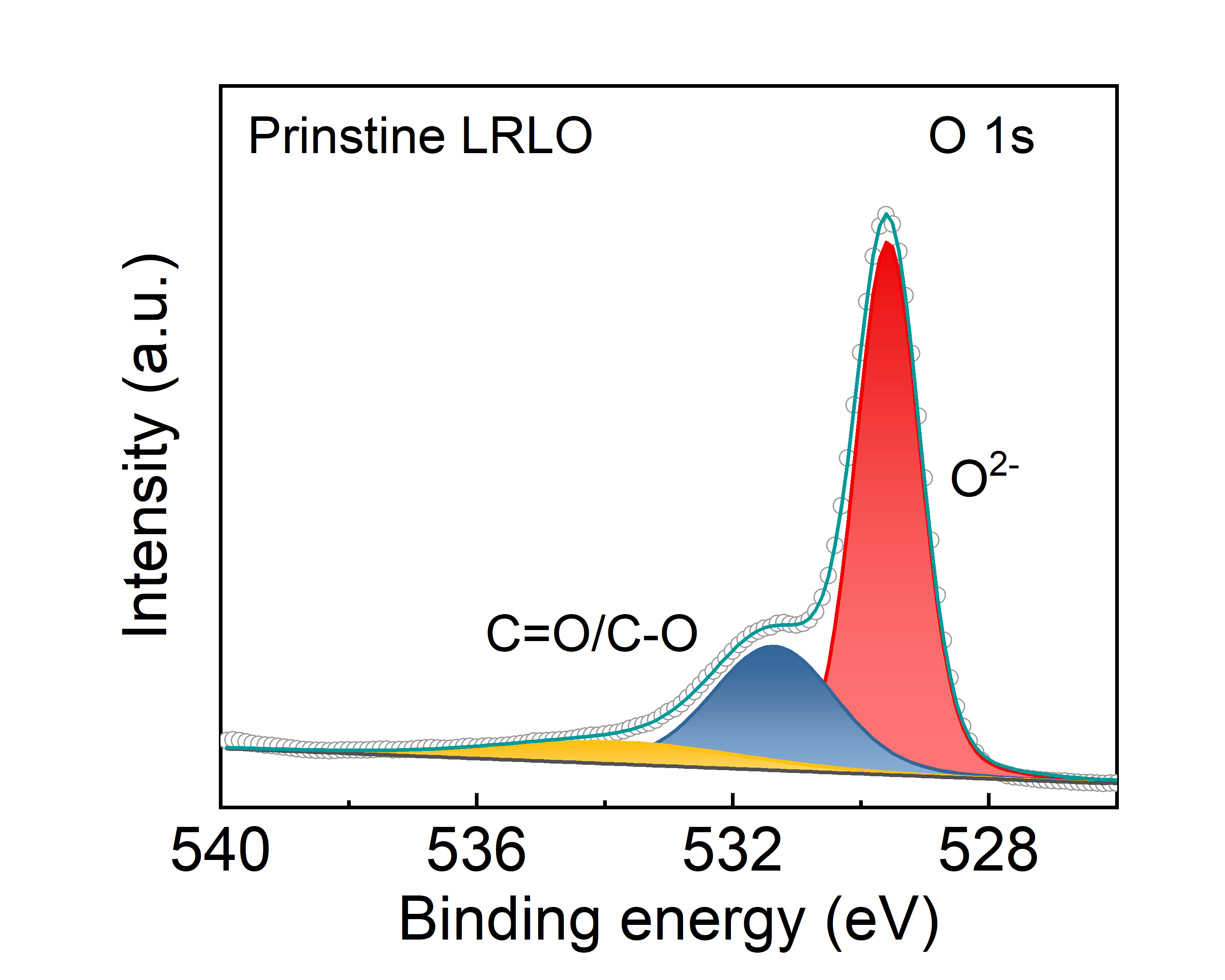


**Figure S4** XPS spectra of O 1s of the pristine LRLO material.


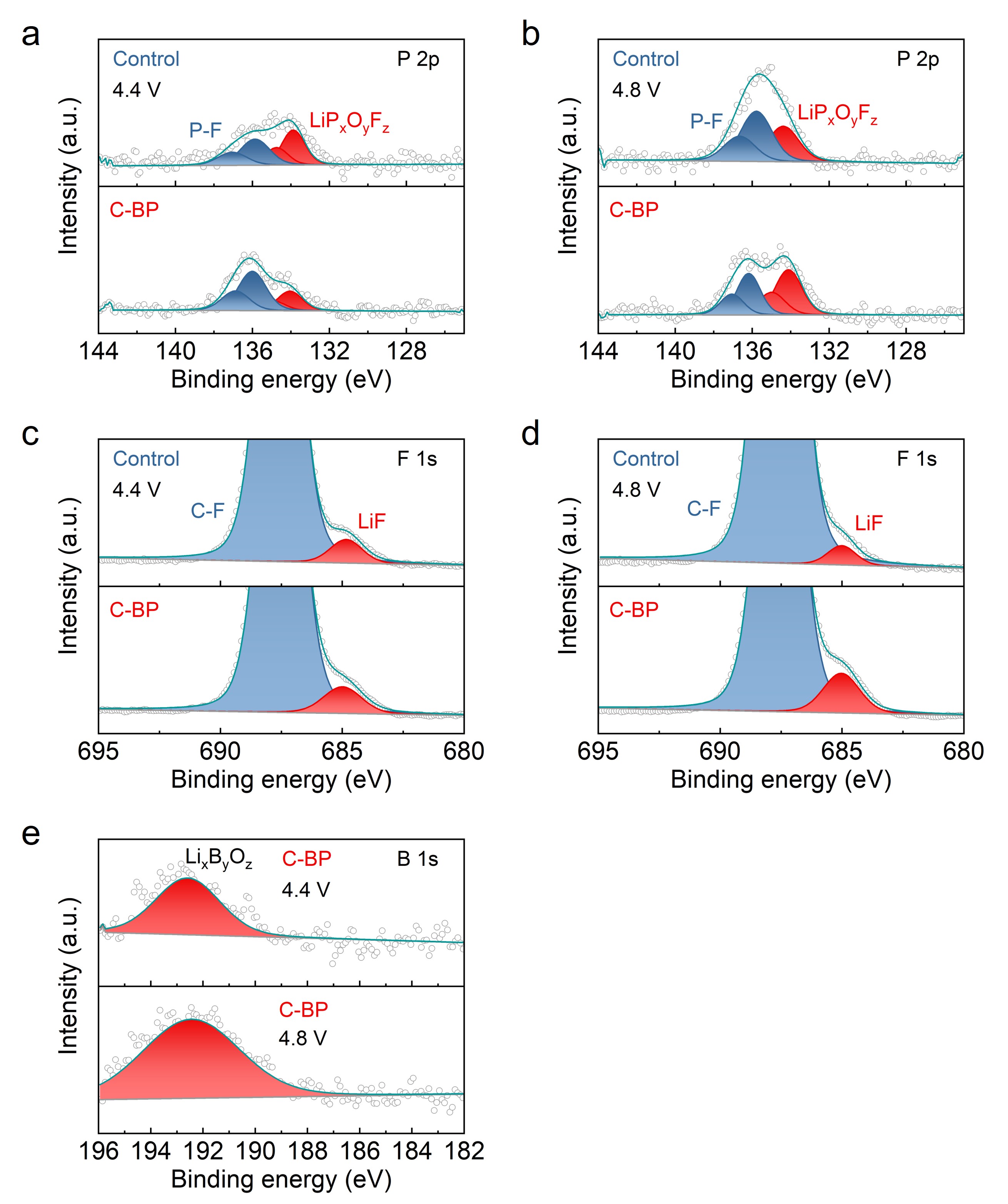


**Figure S5** XPS profiles of (a-b) P 2p, (c-d) F 1s, and (e) B 1s of LRLO cathodes cycled in control and C-BP electrolytes at different cut-off voltages of 4.4 and 4.8 V.


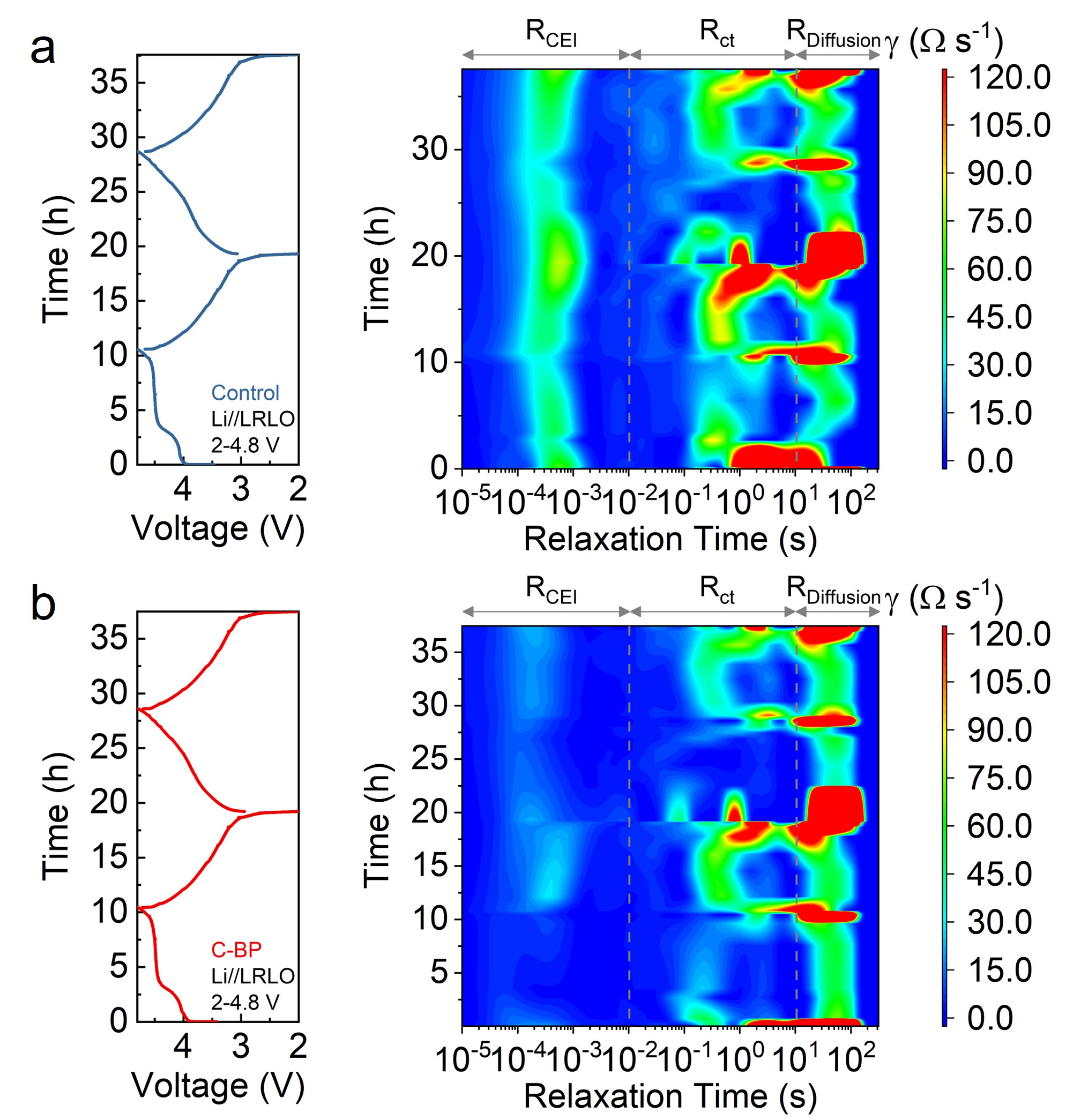


**Figure S6** *In-situ* DRT data of the Li//LRLO cells with (a) control and (b) C-BP electrolytes.


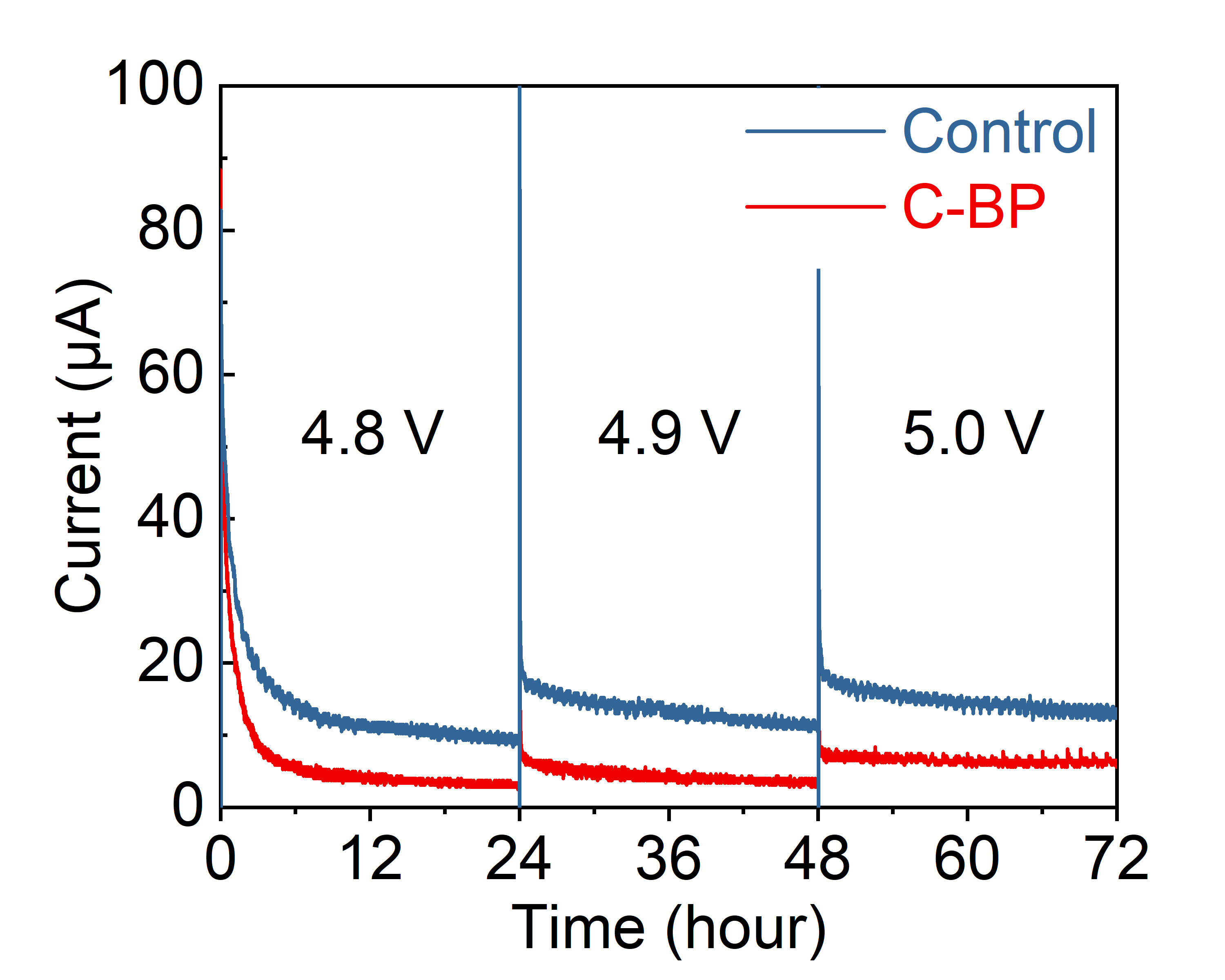


**Figure S****7** Floating charge tests of Li//LRLO cells with control and C-BP electrolytes held at the voltages of 4.8, 4.9, and 5.0 V.


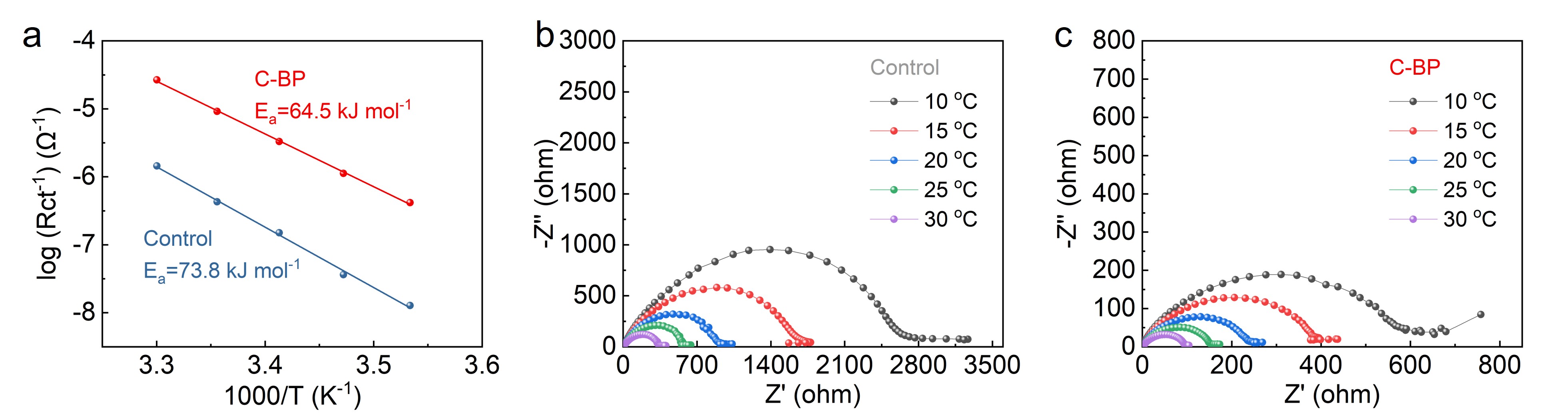


**Figure S8** (a) Li^+^ desolvation activation energy of the control and C-BP electrolytes. Nyquist plots of Li//Li symmetric cells with (b) control and (c) C-BP electrolytes at different temperatures.


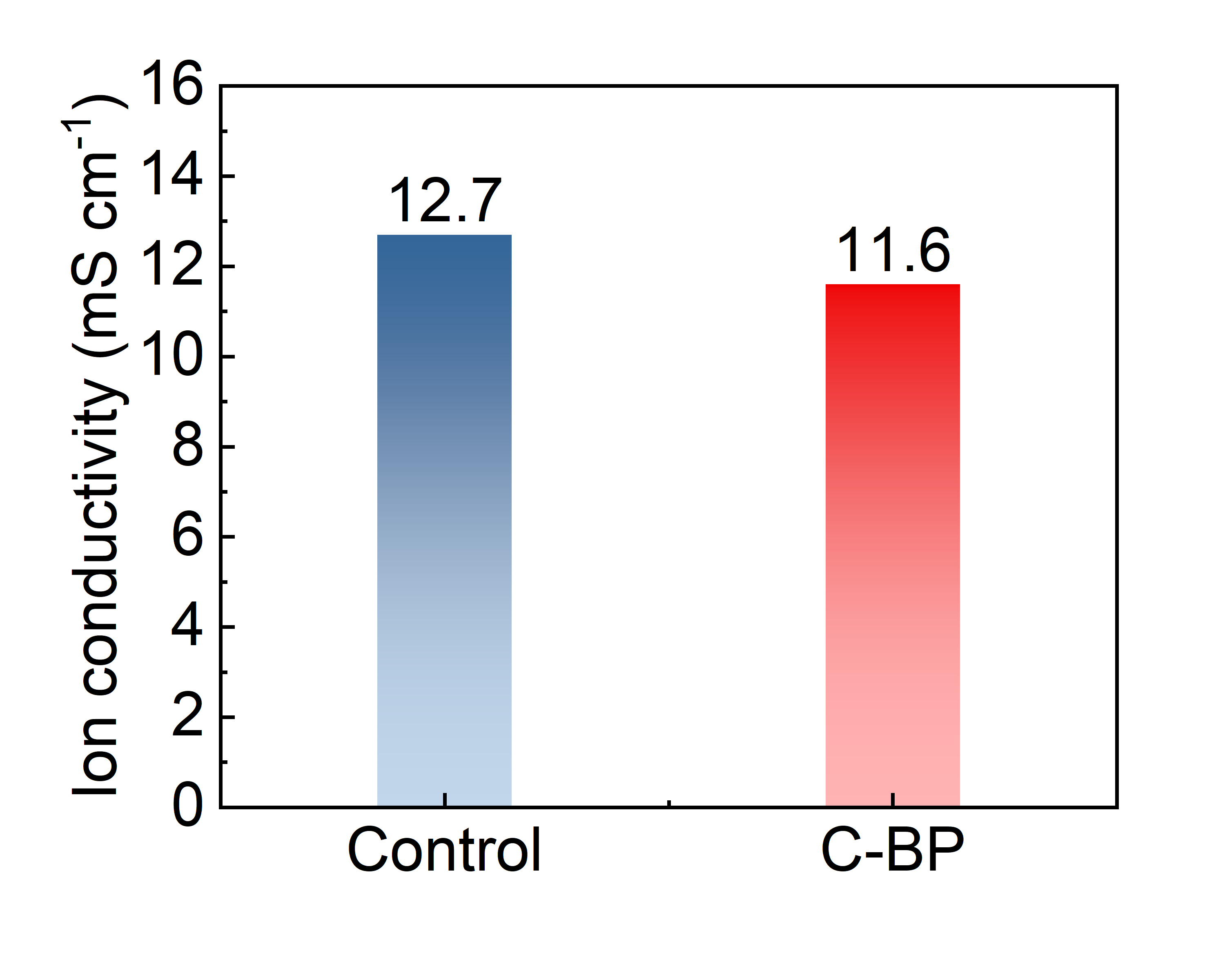


**Figure S9** Ion conductivity of the control and C-BP electrolytes at 30 °C.


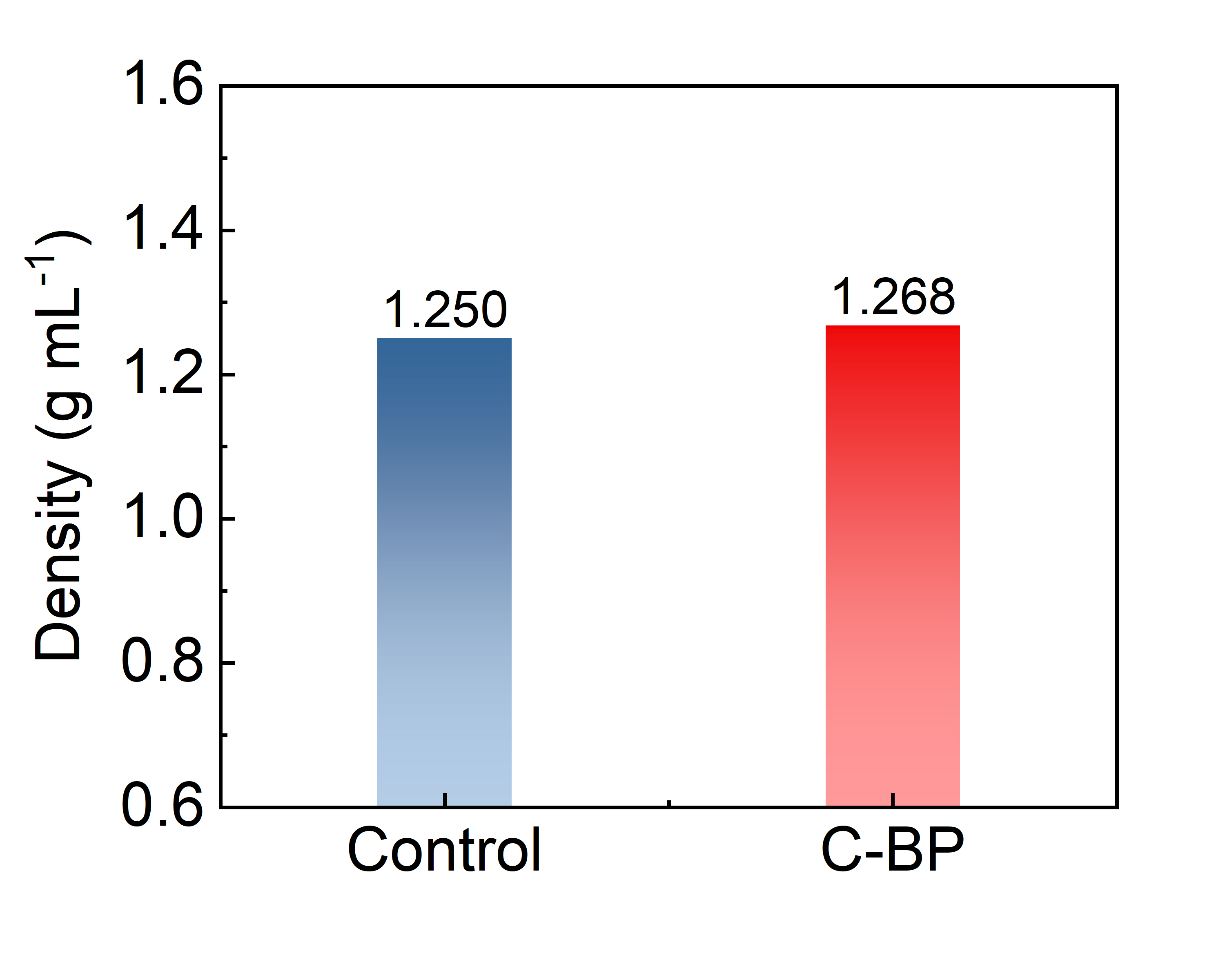


**Figure S10** Density of the control and C-BP electrolytes.


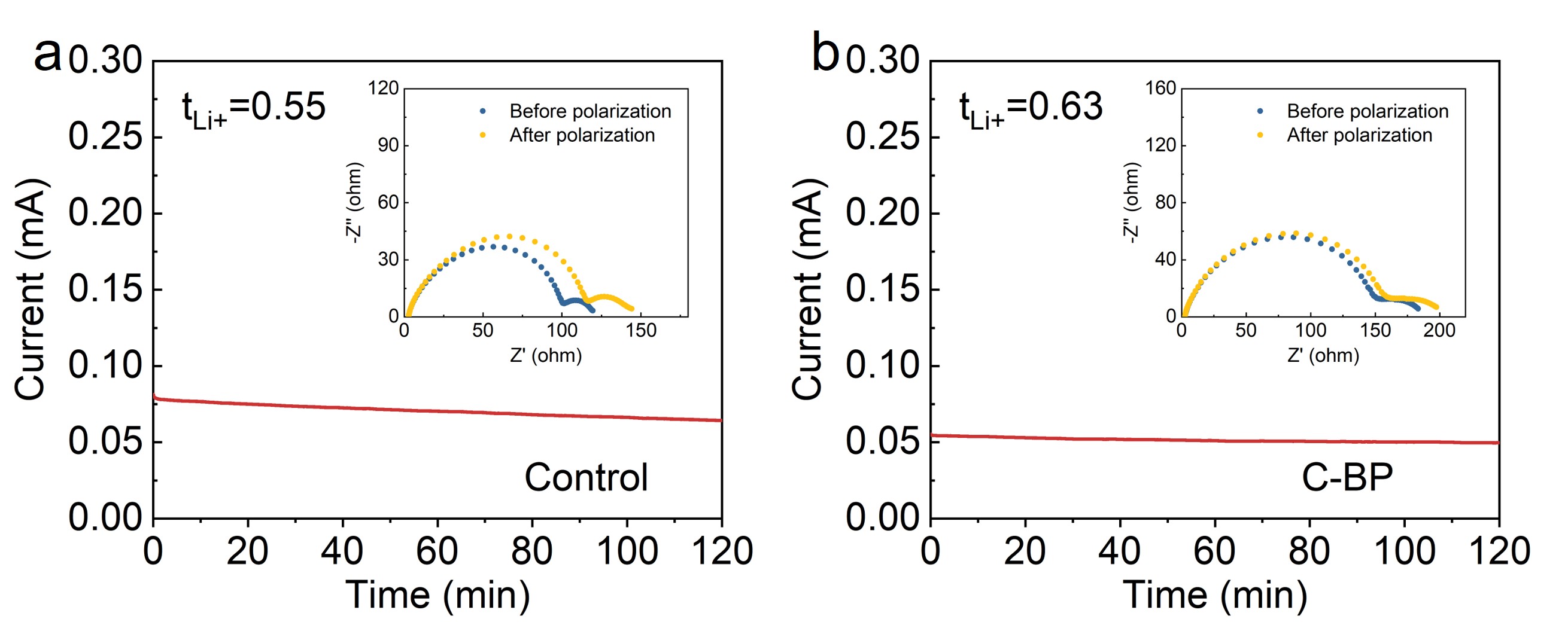


**Figure S11** Chronoamperometry profiles of Li//Li cells using (a) control and (b) C-BP electrolytes and the corresponding Nyquist plots of EIS before and after polarization.


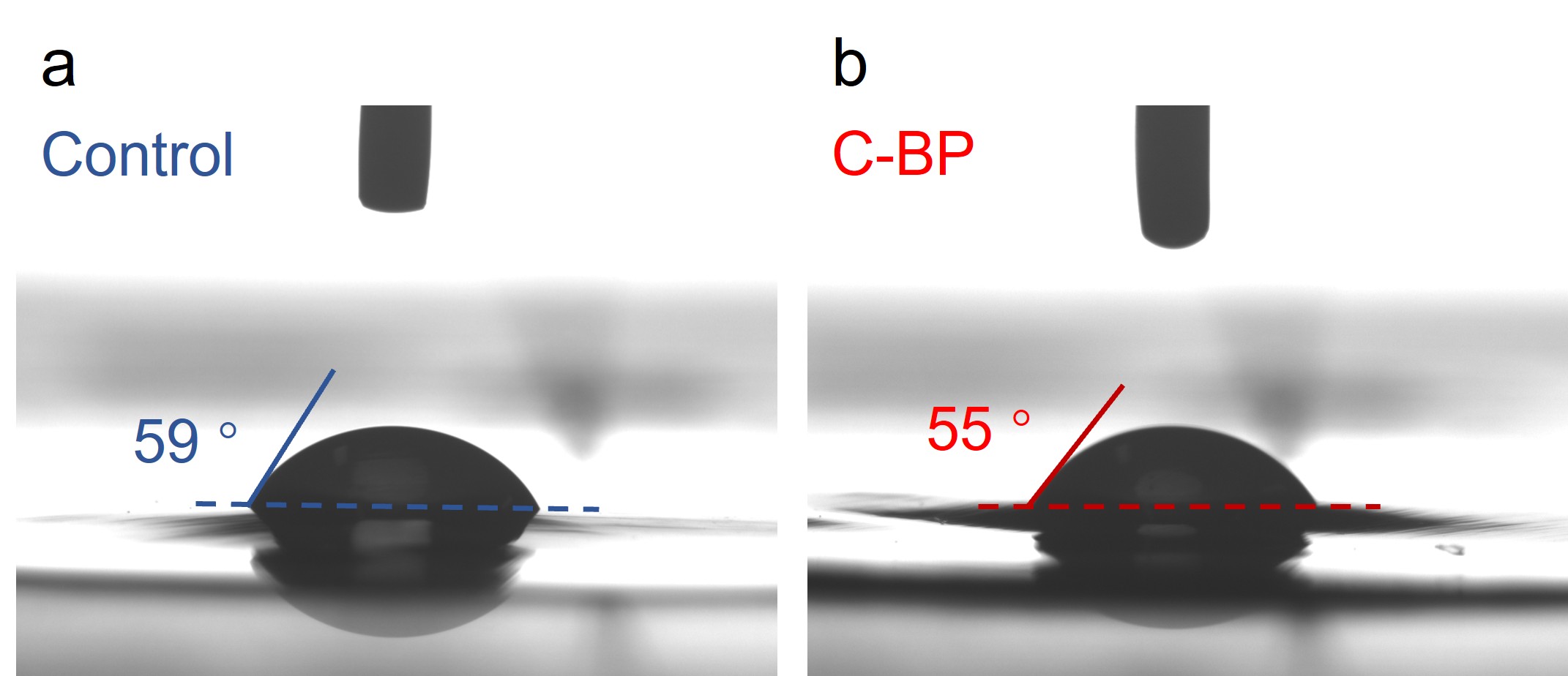


**Figure S12** Wettability of the control and C-BP electrolytes with separator.


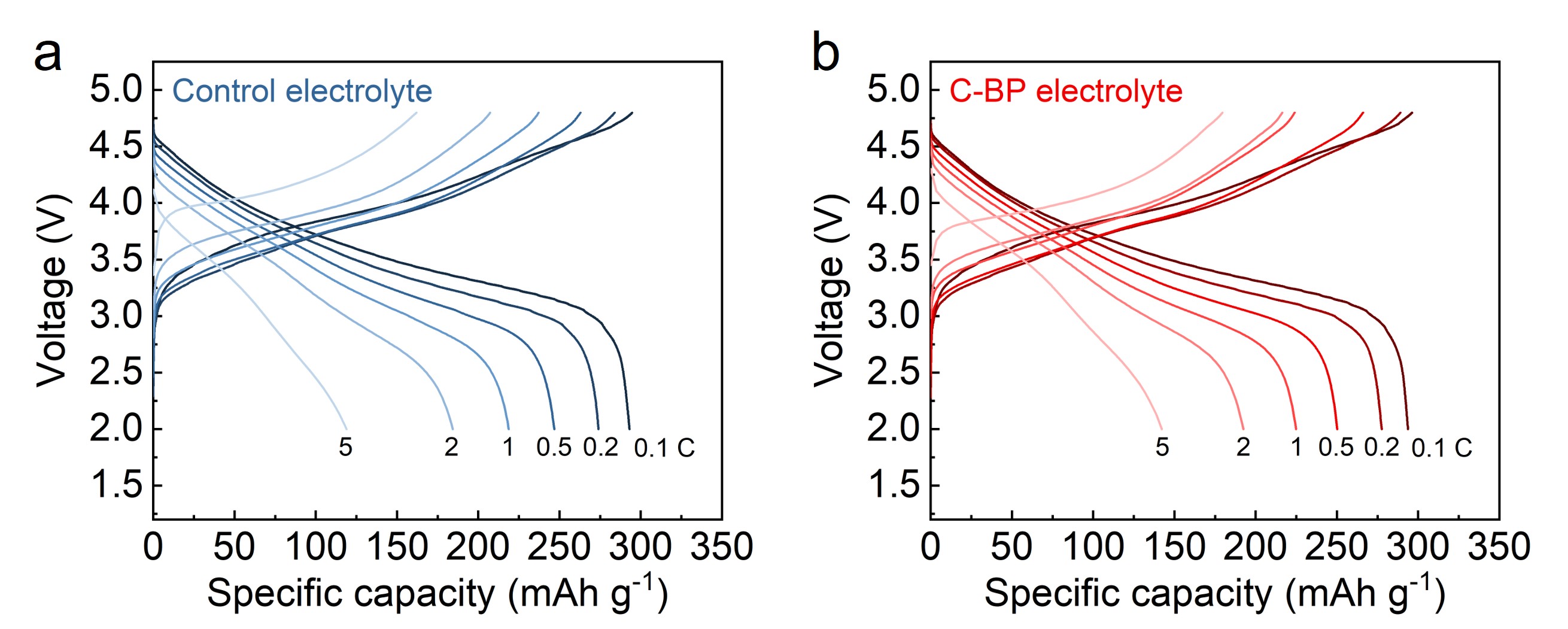


**Figure S13** Charge/discharge curves of Li//LRLO with the (a) control and (b) C-BP electrolytes at different rates and at the temperature of 30 °C.


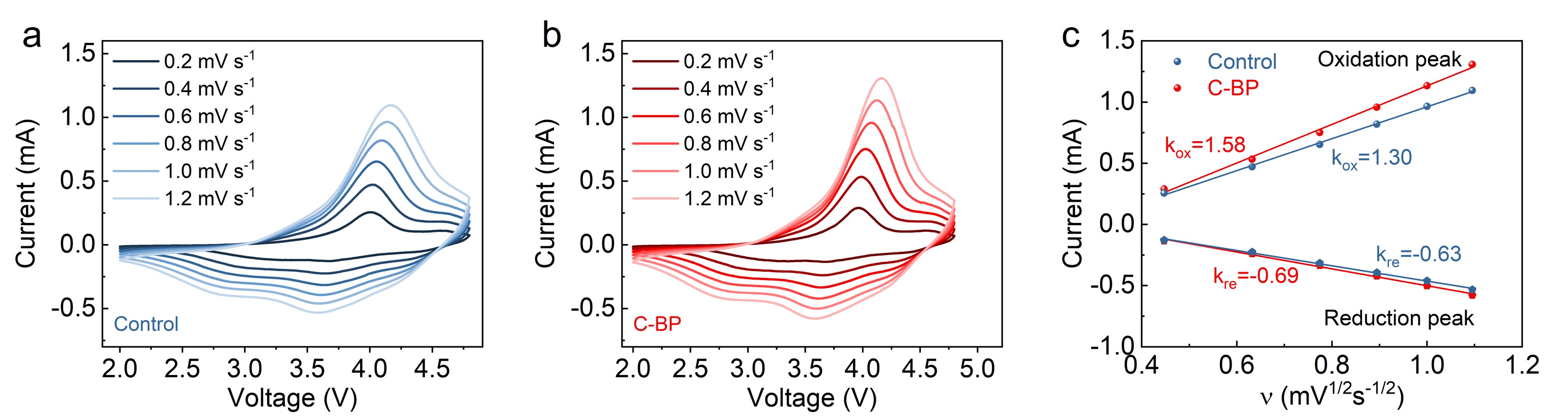


**Figure S14** CV curves of the Li//LRLO cells with (a) control and (b) C-BP electrolytes at different scan rates. (c) The correlation between the peaks of oxidation/reduction and scan rates.


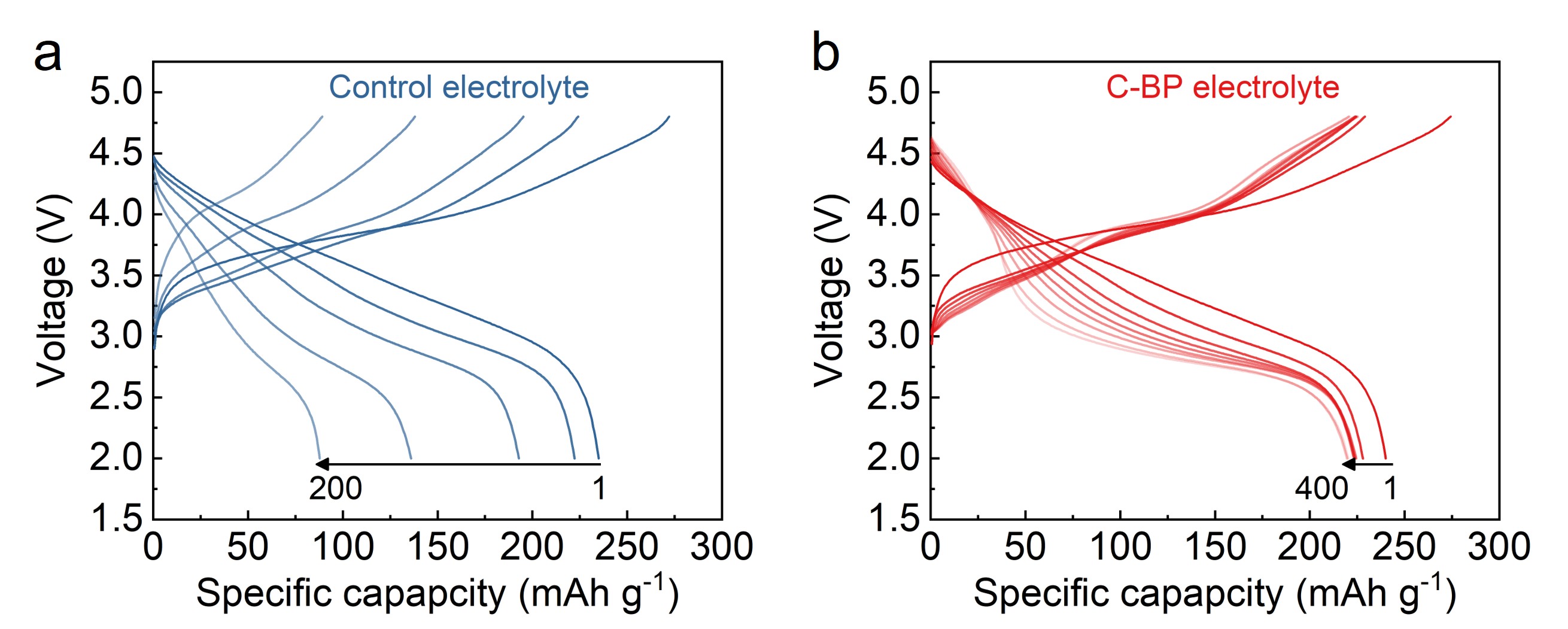


**Figure S15** Charge/discharge curves of the LRLO cathodes cycled in the control and C-BP electrolytes at selected cycles.


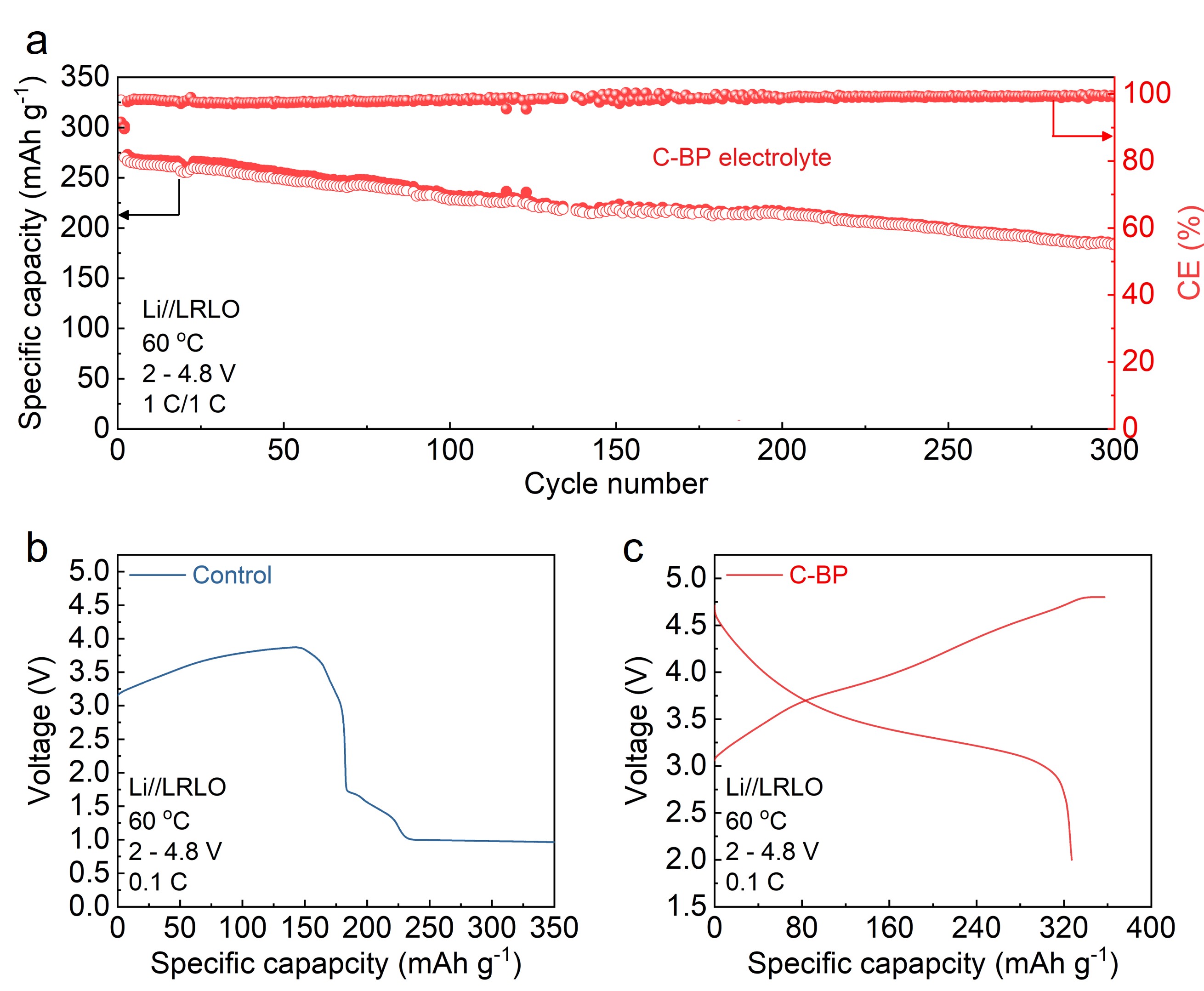


**Figure S16** (a) Cycling performance of Li//LRLO cells in the C-BP electrolytes with the cut-off voltage between 2-4.8 V at the temperature of 60 °C. The corresponding charge/discharge curves of Li//LRLO with the (b) control and (c) C-BP electrolytes at 0.1C.

**Table S1** Literature reviews on high-voltage electrolytes for LRLO cathodes with different mass loadings.

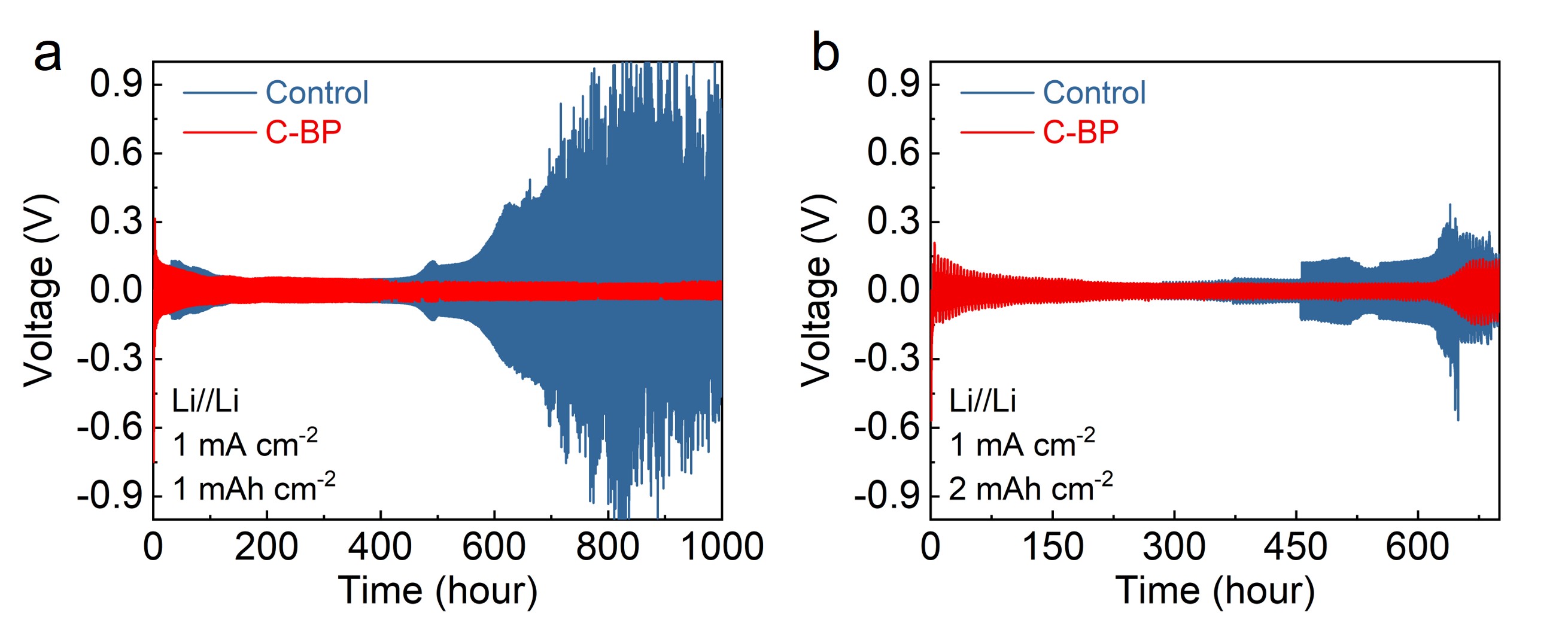


**Figure S17** Cycling stability of Li//Li symmetric cells with control and C-BP electrolytes at the current density of 1 mA cm^-2^ with deposition capacities of (a) 1 mAh cm^-2^ and (b) 2 mAh cm^-2^.


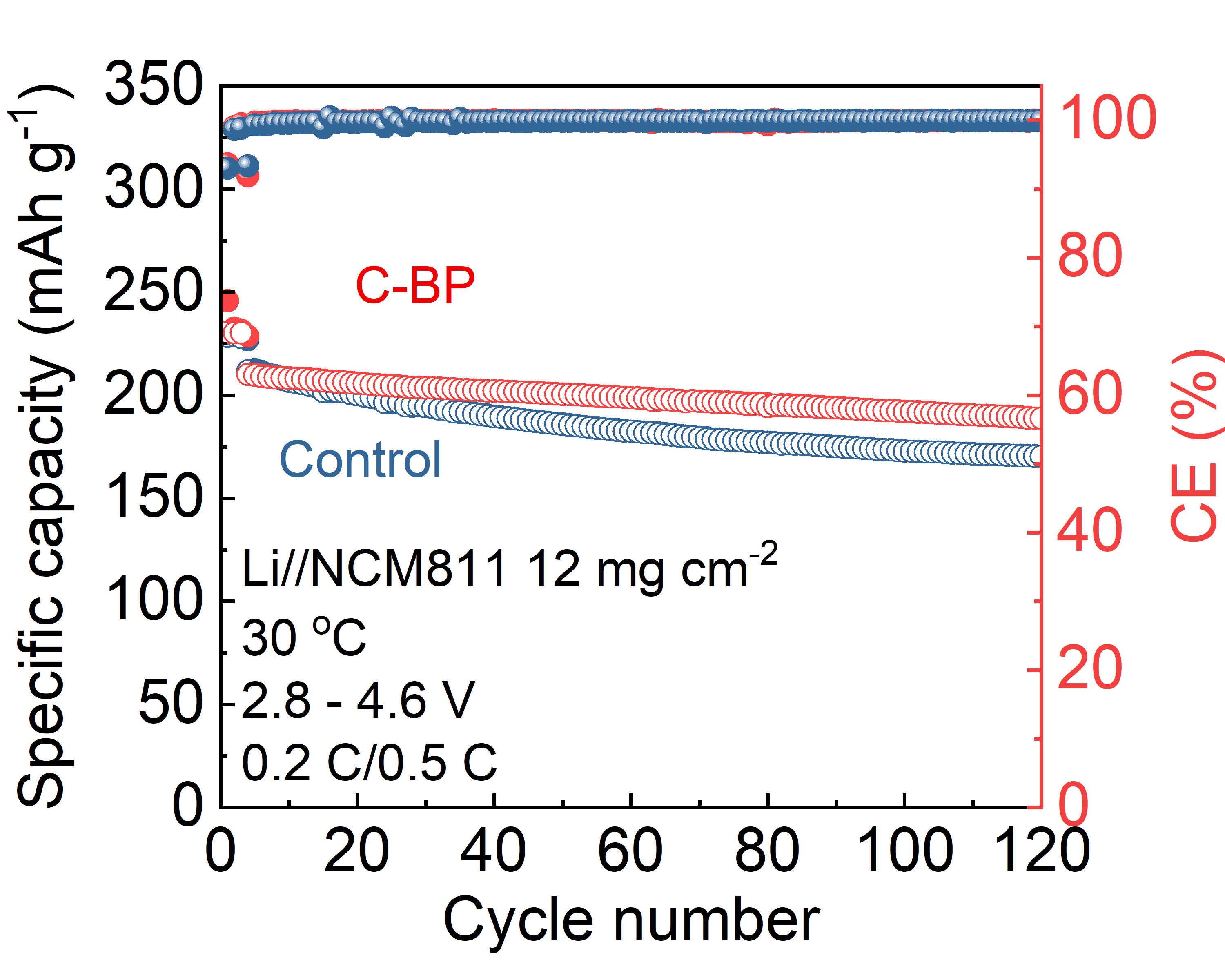


**Figure S18** Electrochemical performance of high mass loading NCM811 (12 mg cm^-2^) with a limited lithium anode (N/P ratio=3.8) in control and C-BP electrolytes at 0.2C charge/0.5C discharge.


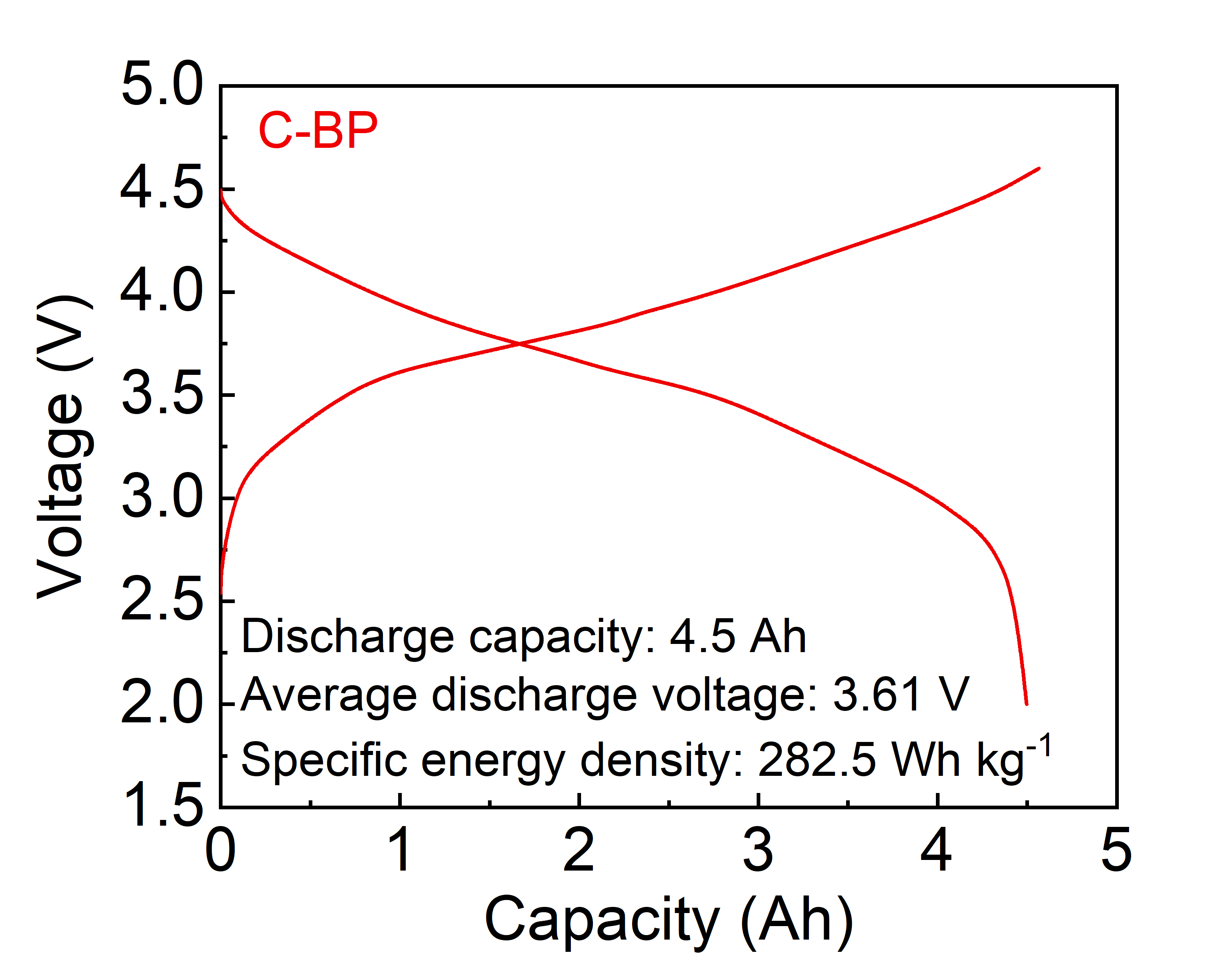


**Figure S19** Typical charge/discharge curves of the graphite//LRLO pouch cell with the C-BP electrolyte, cycled at 0.1 C within the electrochemical window of 2-4.6 V.


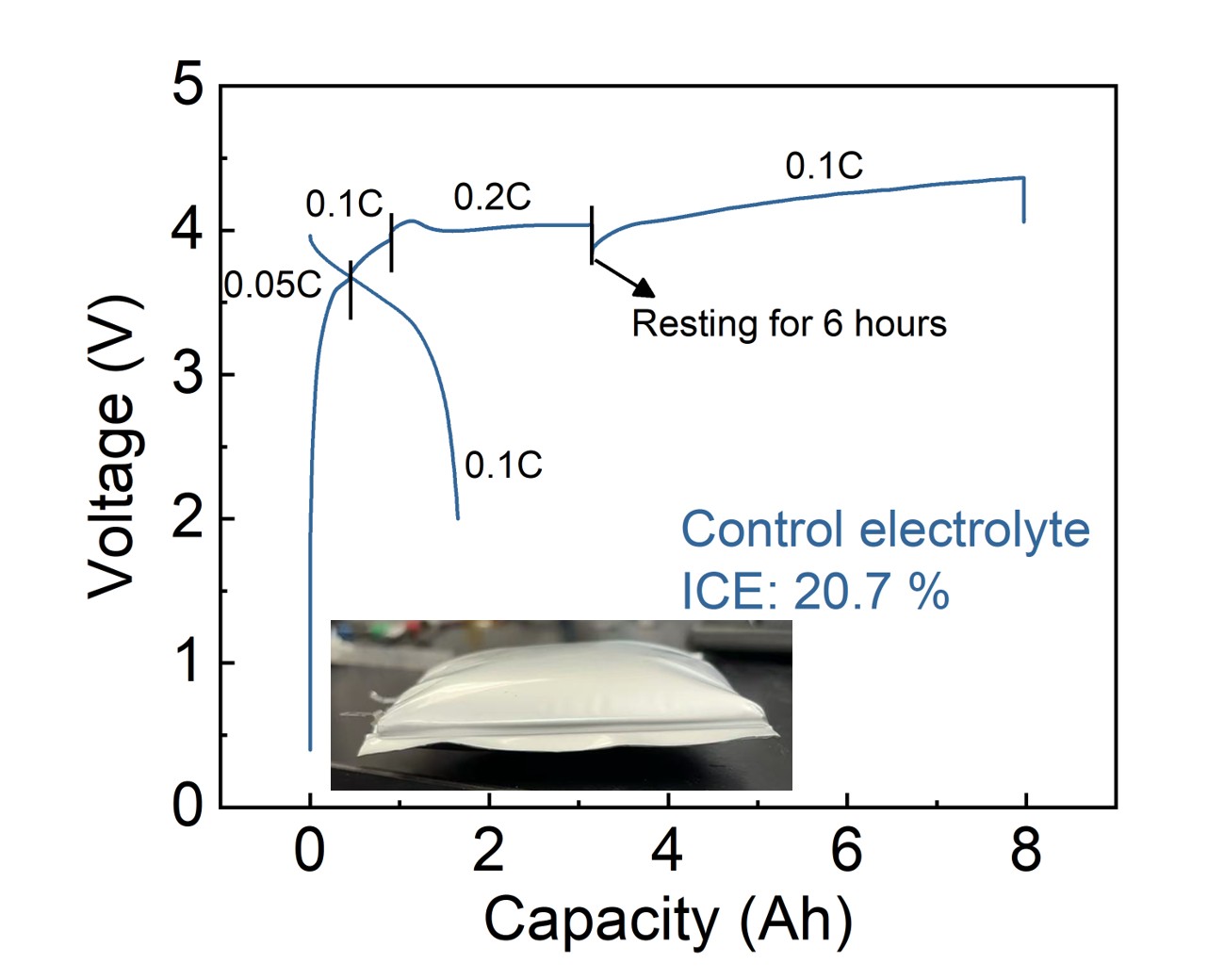


**Figure S20** the initial charge/discharge curves of the graphite//LRLO pouch cell with control electrolyte (insect: digital photo of the pouch cell after initial cycle).


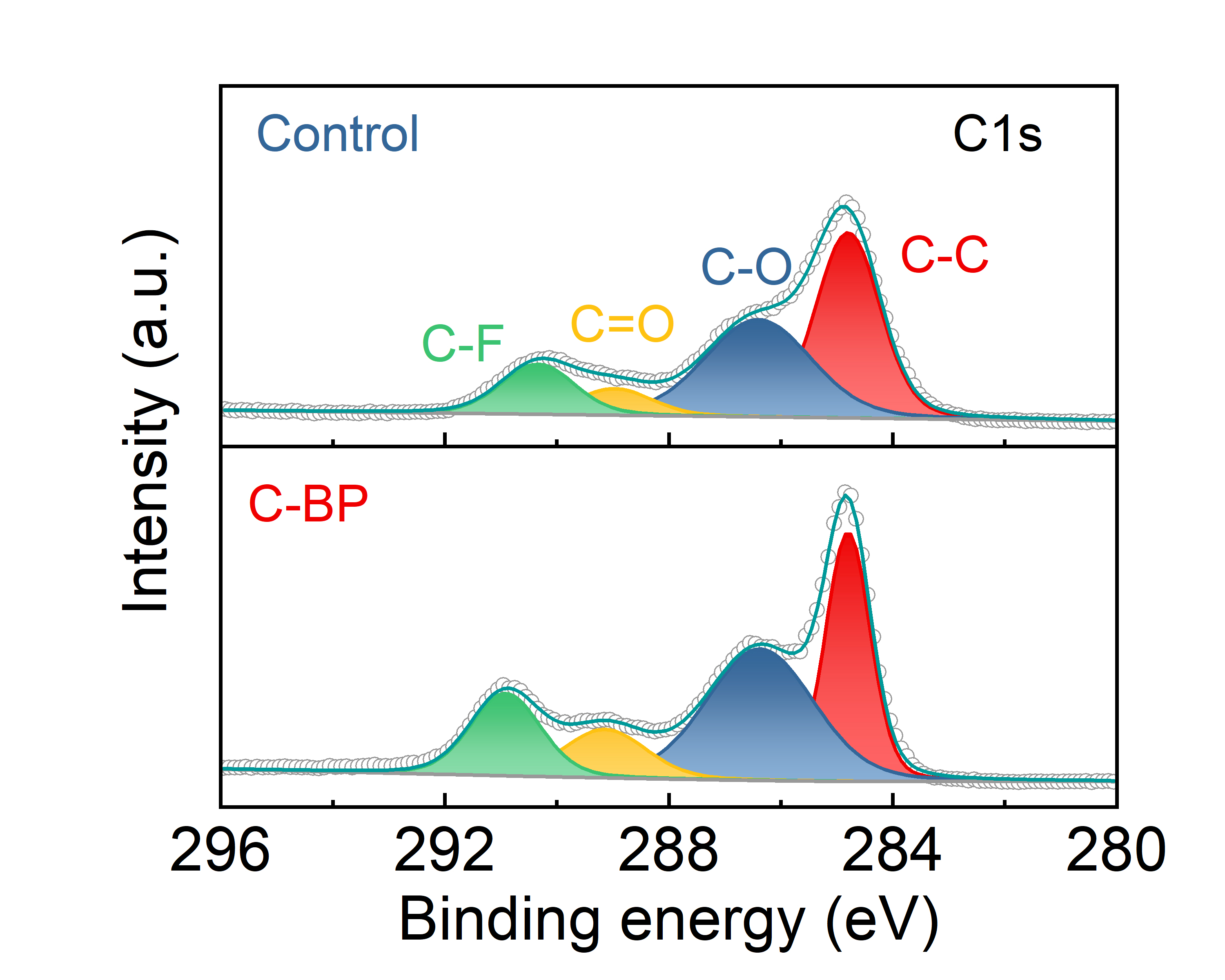


**Figure S21** XPS spectra of C 1s of the LRLO cathodes cycled in the control and C-BP electrolytes for 50 cycles.


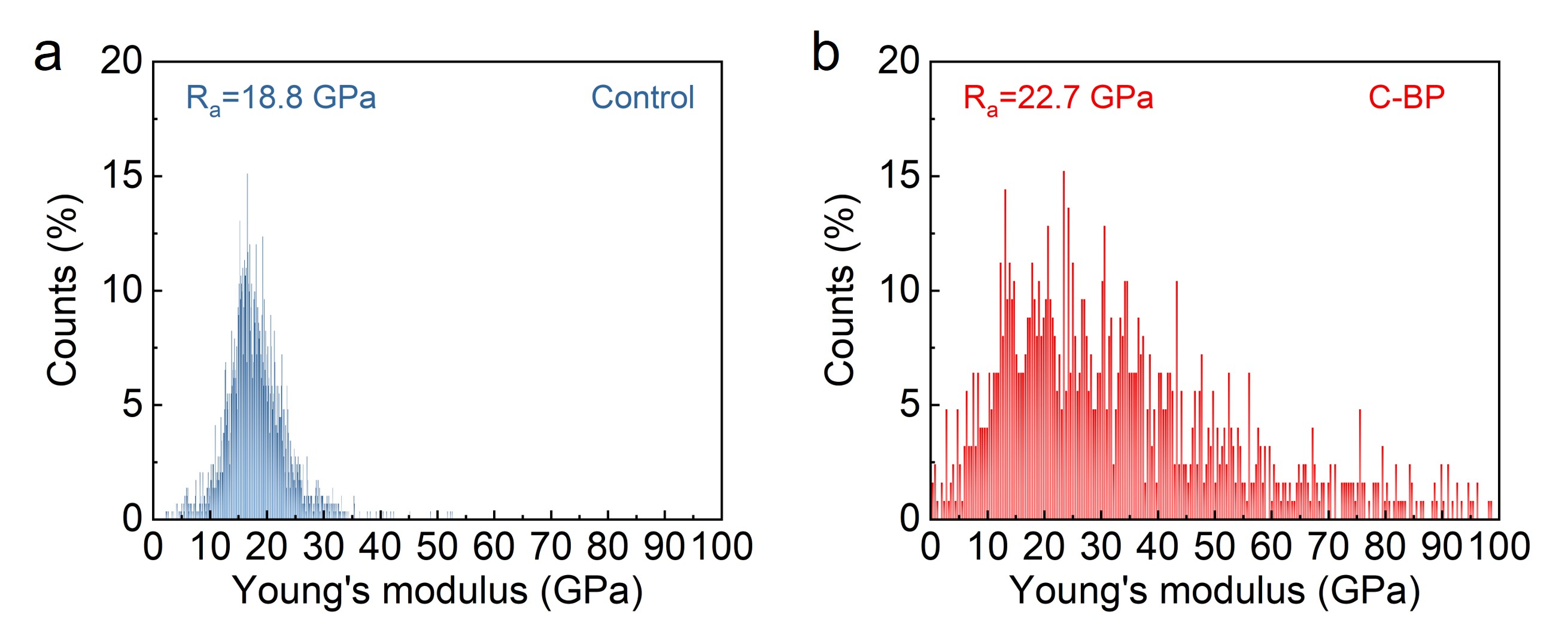


**Figure S22** Young’s modulus contribution of LRLO cathodes cycled in the control and C-BP electrolytes obtained from AFM tests.


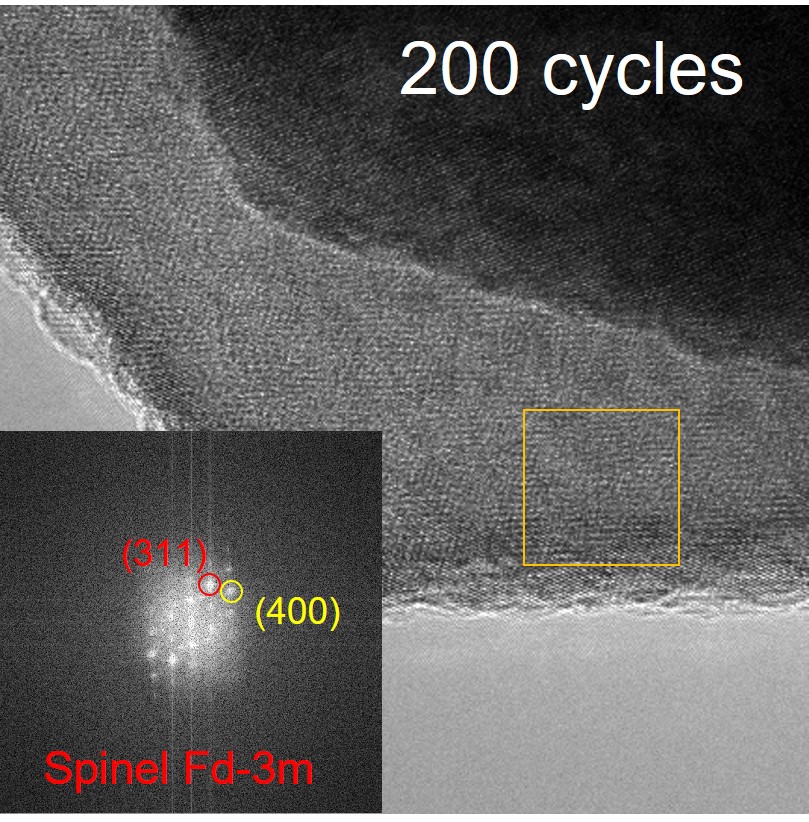


**Figure S23** HR-TEM images of the LRLO particles in control electrolyte after 200 cycles.


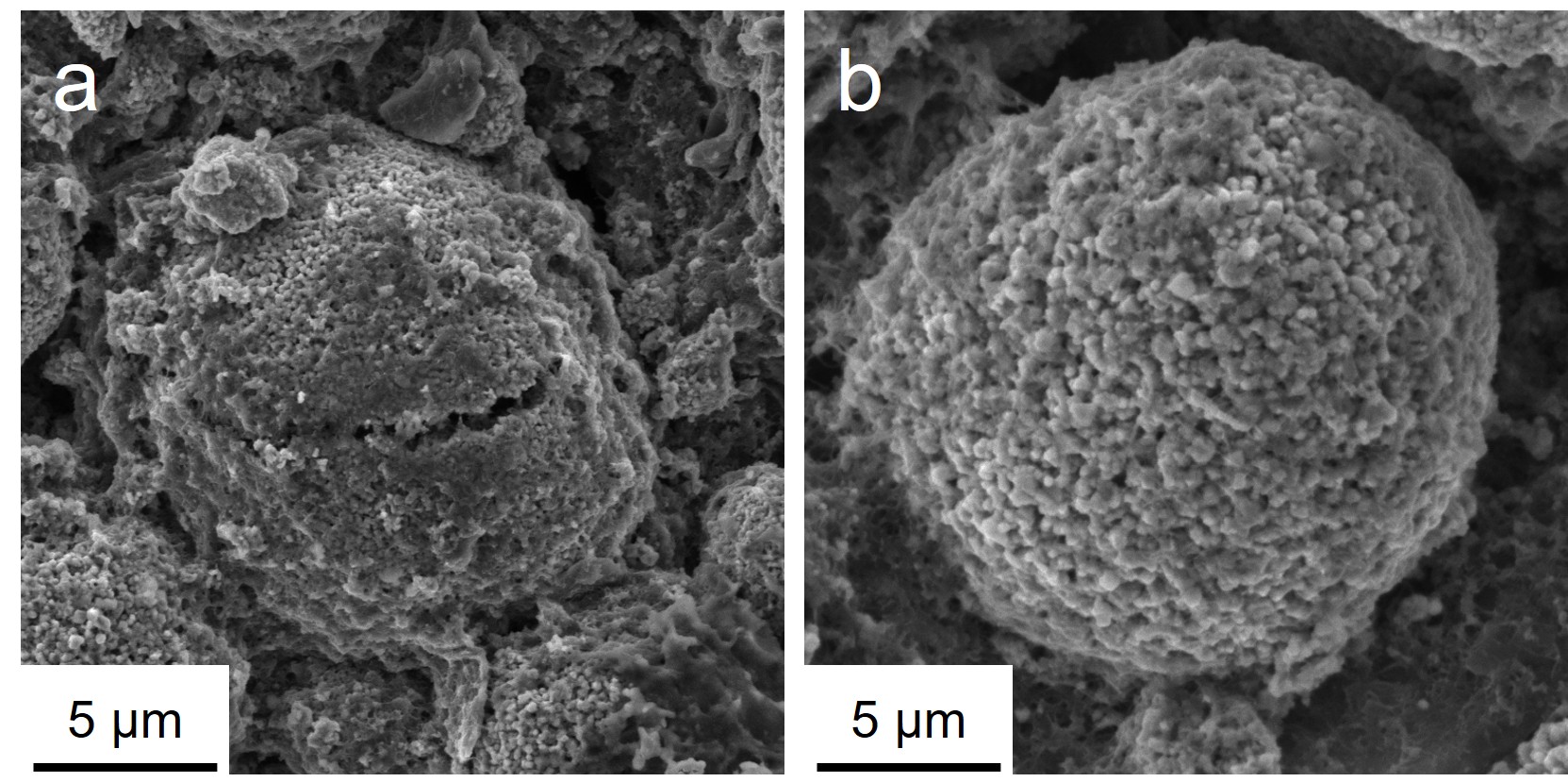


**Figure S24** SEM images of the LRLO particle cycled in (a) control and (b) C-BP electrolytes after 200 cycles.


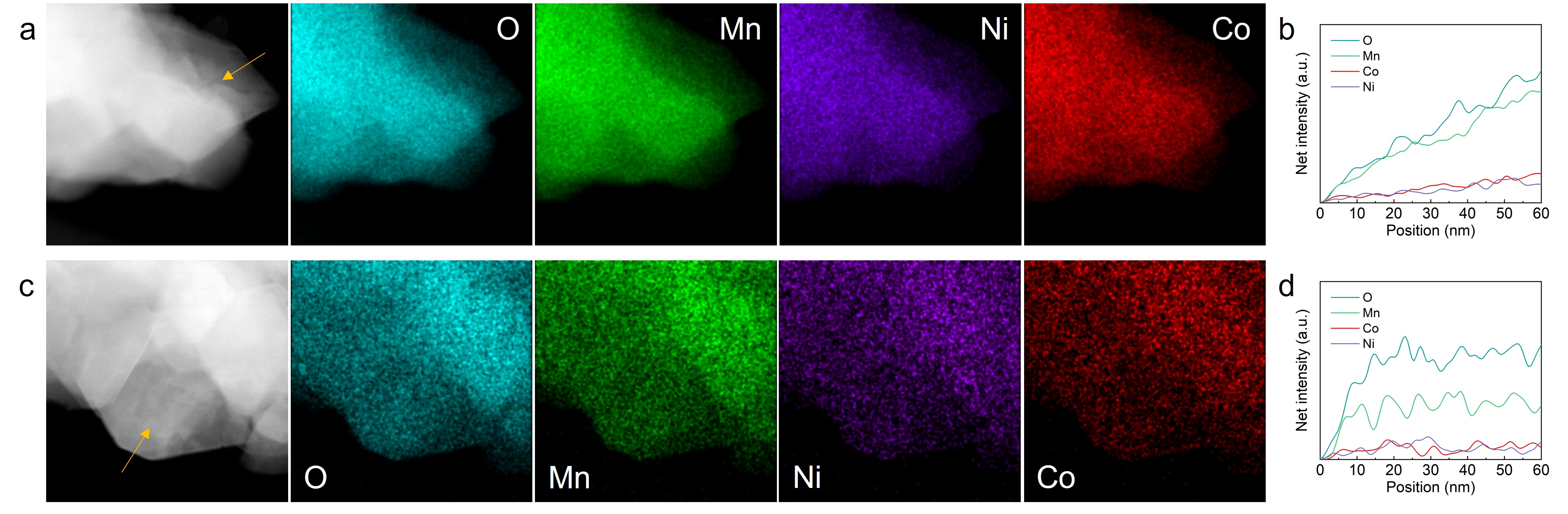
**Figure S25** HAADF-STEM images and the corresponding EDS elemental mapping images of LRLO particles cycled in (a) control and (c) C-BP electrolytes. The line scanning of EDS mapping along the yellow line of the HAADF-STEM images of the LRLO particle cycled in (b) control and (d) C-BP electrolytes.


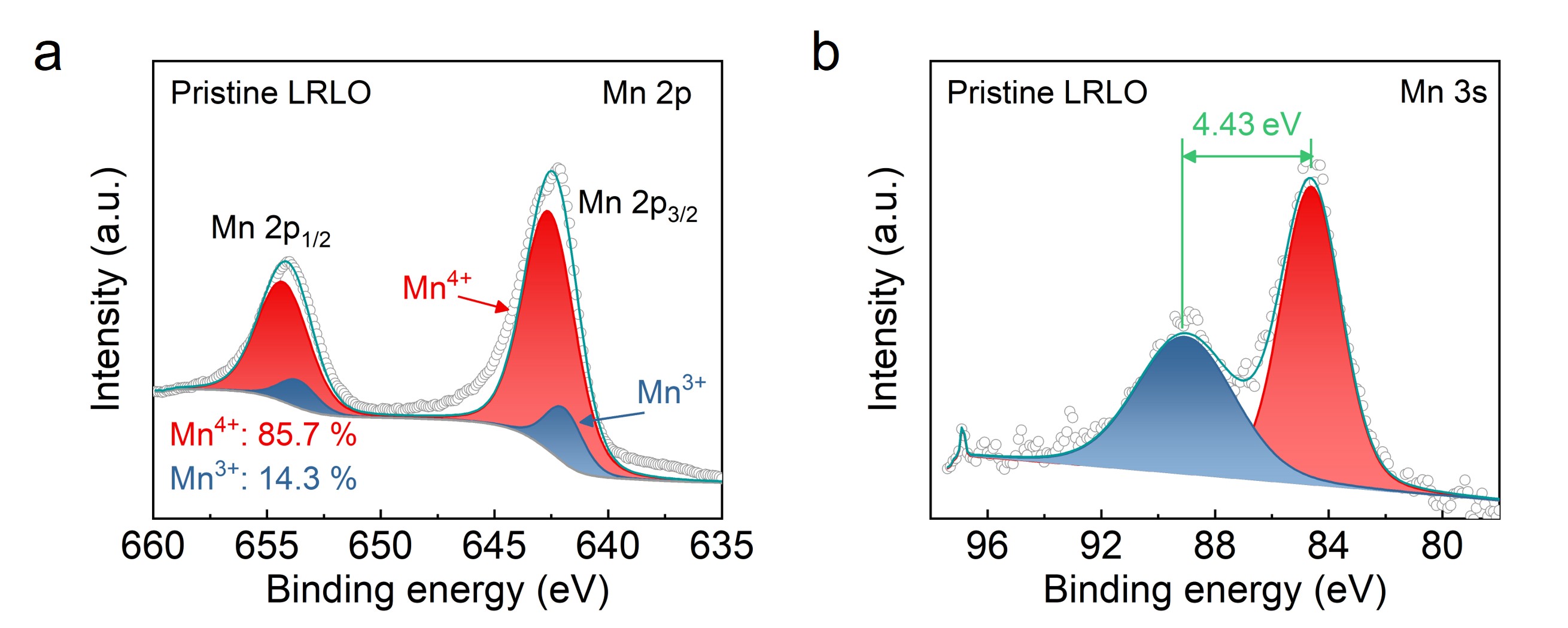


**Figure S26** XPS spectra of (a) Mn 2p and (b) Mn 3s of the pristine LRLO material.


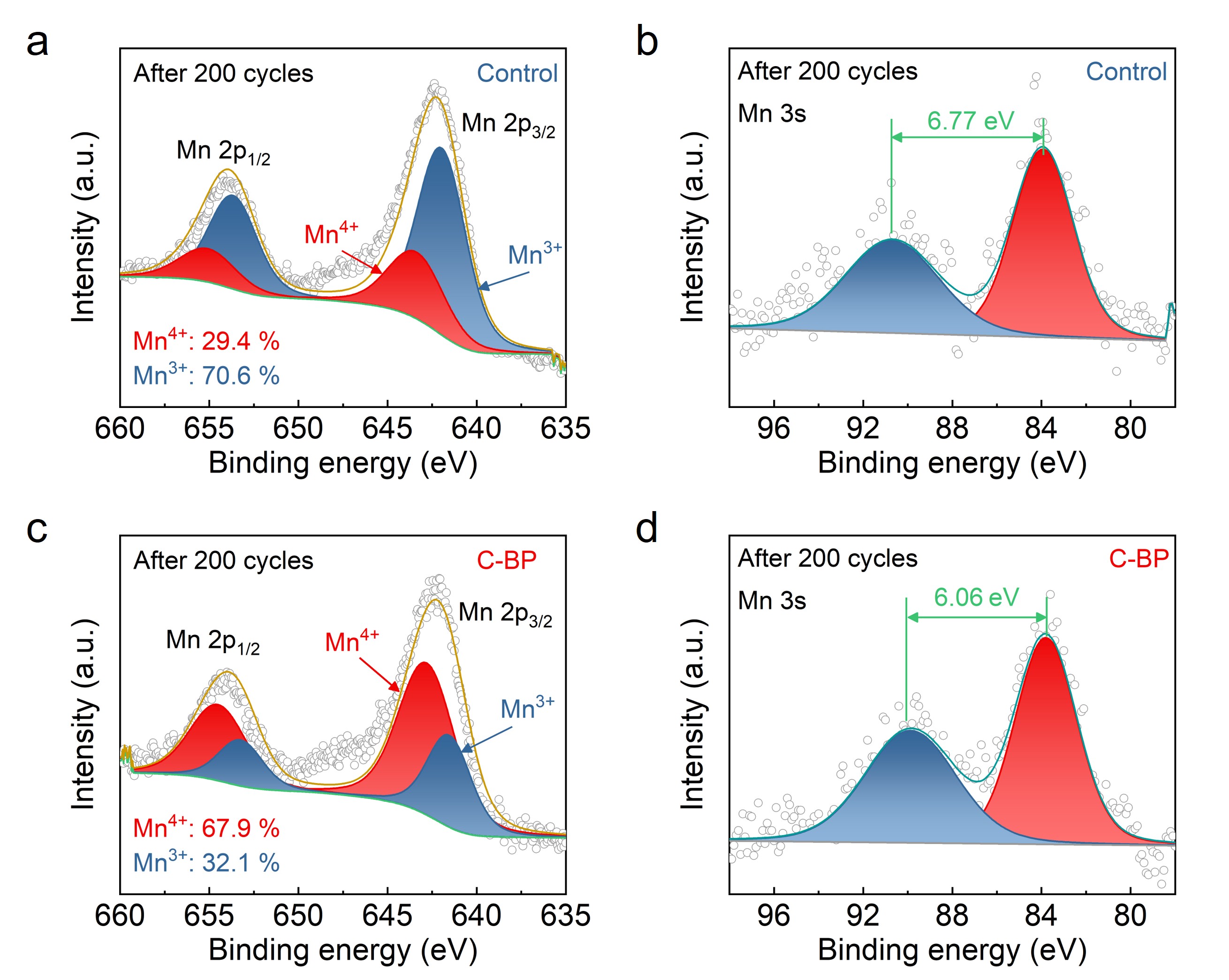


**Figure S27** XPS spectra of Mn 2p and Mn 3s of the cycled LRLO cathodes in the (a-b) control and (c-d) C-BP electrolytes after 200 cycles at 1C.


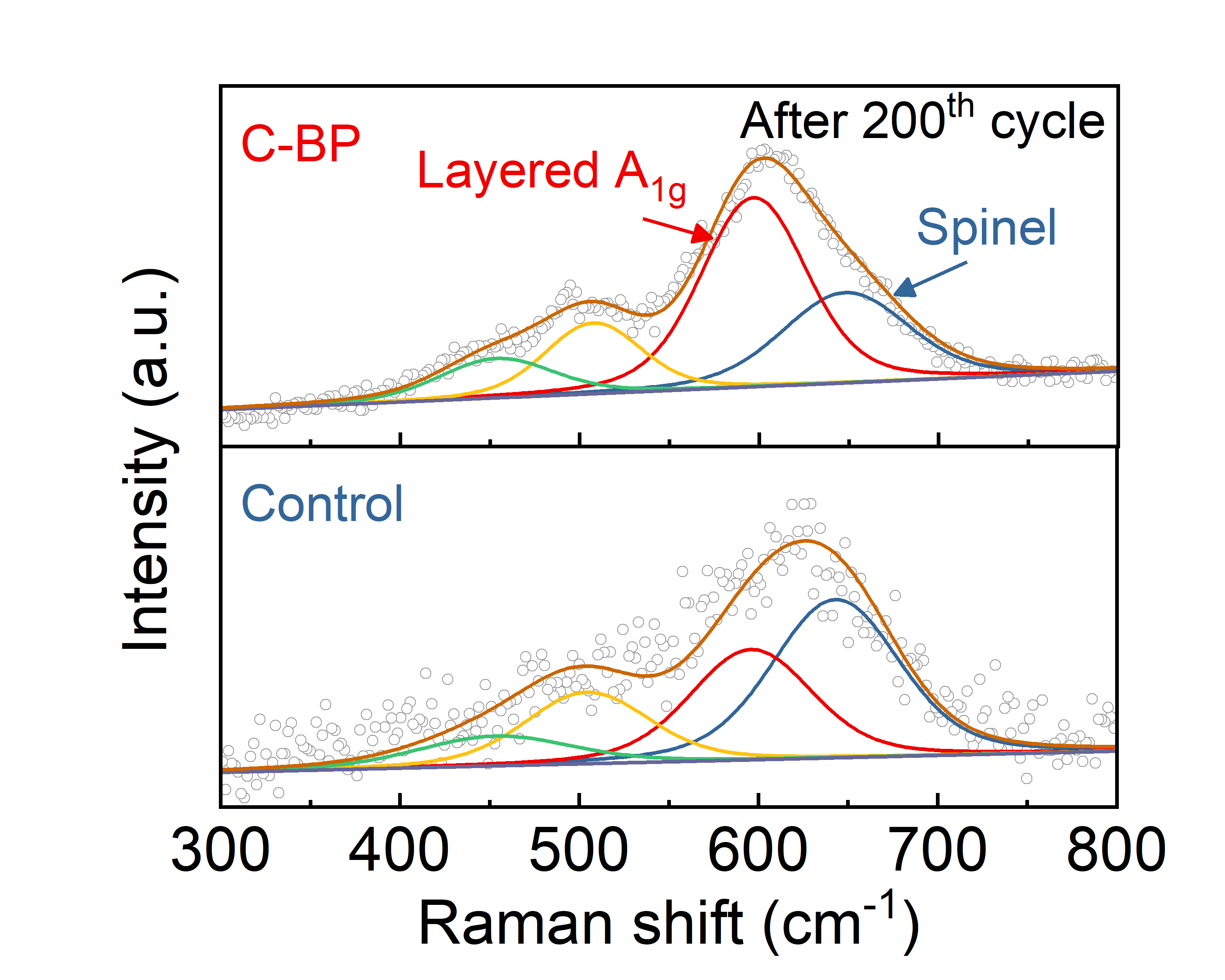


**Figure S28** Raman spectra of the LRLO cathodes cycled in the control and C-BP electrolytes after 200 cycles.

**Reference:**

[1] T. H. Wan, M. Saccoccio, C. Chen, F. Ciucci, *Electrochim. Acta* **2015**, *184*, 483.

[2] Y. Lu, C.-Z. Zhao, J.-Q. Huang, Q. Zhang, *Joule* **2022**, *6*, 1172.

[3] Z. Wang, Y. Wang, B. Py, A. Maradesa, J. Liu, T. H. Wan, M. Saccoccio, F. Ciucci, *ACS Electrochem.* **2025**, *1*, 2680.

[4] T. Clark, J. Chandrasekhar, G. W. Spitznagel, P. V. R. Schleyer, *J. Comput. Chem.* **1983**, *4*, 294.

[5] F. Weigend, R. Ahlrichs, *Phys. Chem. Chem. Phys.* **2005**, *7*, 3297.

[6] S. Zhang, S. Li, X. Wang, C. Li, Y. Liu, H. Cheng, S. Mao, Q. Wu, Z. Shen, J. Mao, H. Pan, Y. Lu, *Nano Energy* **2023**, *114*, 108639.

[7] M. Frish, G. Trucks, H. Schlegel, G. Scuseria, M. Robb, J. Cheeseman, G. Scalmani, V. Barone, B. Mennucci, G. Petersson, Other, Gaussian 09, revison D. 01. In Gaussian, Inc., Wallingford CT: 2009.
